# Excited State Dynamics of CO_2_ Reduction Catalyst under Vibrational Strong Coupling

**DOI:** 10.1021/jacs.5c11060

**Published:** 2025-10-10

**Authors:** Tao Jin, Sara T. Gebre, Christopher J. Miller, Clifford P. Kubiak, Raphael F. Ribeiro, Tianquan Lian

**Affiliations:** † Department of Chemistry, 1371 Emory University, 1515 Dickey Drive, Northeast, Atlanta, Georgia 30322, United States; ‡ Department of Chemistry and Biochemistry, 8784 University of California San Diego, 9289 S Scholars Dr, La Jolla, California 92093, United States; § Department of Chemistry, 6572 University of Pennsylvania, 231 S 34th Street, Philadelphia, Pennsylvania 19104, United States; # Department of Physics, 1371 Emory University, 1515 Dickey Drive, Northeast, Atlanta, Georgia 30322, United States

## Abstract

Molecular polaritons, formed by coupling molecular electronic or vibrational transitions to photonic modes in microcavities, have gained interest for their potential to influence chemical dynamics. Here, we investigate the effects of vibrational strong coupling (VSC) on solvation-induced time-dependent Stokes shifts using transient infrared (IR) transmission spectroscopy. The electronic excited-state dynamics of the Re­(bpy-COOH)­(CO)_3_Cl complex (ReC0A) is monitored via angle-resolved time-dependent transmission spectra of vibrational polaritons following 400 nm excitation inside a Fabry–Perot cavity. Our results reveal distinct infrared polaritonic signatures of the CO dynamical Stokes shift, which we interpret using simulations based on a time-dependent excited-state absorption model. We observed negligible change of the solvation-induced vibrational dynamic Stokes shift of the CO modes under VSC. We also investigate the perturbed free induction decay in the cavity and its connection to polariton dynamics. This setup allows us to probe and test potential fundamental VSC effects on molecular processes relevant to the reactivity and charge transfer.

## Graphical abstract

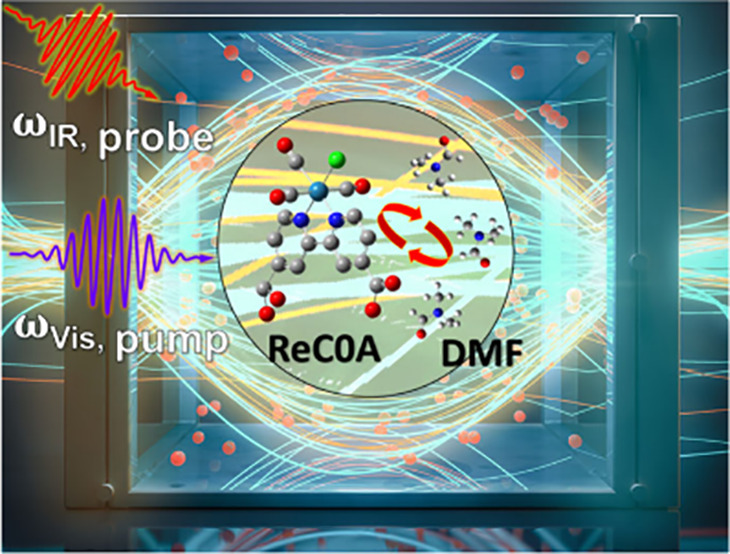

## Introduction

Polaritons are hybrid quasiparticles that emerge from strong light-matter interactions and retain both photonic and matter properties. Polaritons have been widely studied in lasing,^1,2^ condensation,^3^ superfluidity,^4^ and more.^5–8^ More recently, molecular vibrational polaritons, formed by strong coupling of molecular vibrational transitions to the electromagnetic modes of a moderate Q-factor etalon, have received intense interest for applications in chemistry. It has been reported that vibrational strong coupling (VSC) can change reaction product distributions and thus offer a potential novel approach for controlling chemical reactions.^9,10^ Experimental and theoretical studies have shown that molecular polaritons can affect various chemical reactivities,^11–21^ transport and conductivity,^22,23^ intramolecular and intermolecular electron/energy transfer,^23–34^ and molecular nonlinear absorption and relaxation dynamics.^35–41^ However, there has been controversy, as some earlier observations were not reproduced in recent experiments,^42,43^ and a few studies suggest that VSC has a negligible effect on certain reactions^44,45^ and on the intrinsic optical nonlinearity of materials.^46^ These disagreements indicate that while significant progress has been made, many questions remain unanswered and systematic experimental studies probing various aspects of polariton chemistry are needed to advance this field.

Recent studies suggest VSC can be employed to control solvent–solute interactions.^17,47–51^ For instance, recent research has reported that VSC can significantly alter ionic conductivity by modifying the dynamic hydration structure of water molecules under vibrational strong coupling, leading to enhanced ionic conductivity for certain cations.^49,50^ Additionally, an initial report claimed that VSC modulates solvent polarity, impacting solvation dynamics and molecular interactions.^51^ However, this work was later retracted; subsequent analysis showed the apparent shifts arose from Fabry–Pérot interference and cavity-length nonuniformity, rather than VSC.^52^ Due to this, the concept of modifying solvation properties under VSC remains unestablished and requires stringent controls. This underscores the importance of careful experimental design and interpretation in future efforts to explore the solvation effects in vibrational polaritonic systems.

In this work, we pursue a new route to investigate vibrational strong coupling effects on solvent response and excited state dynamics, as these processes play crucial roles in thermal and photoinduced chemical reactions. These aspects have attracted significant theoretical interest, with predictions indicating that these dynamics could be modified by VSC.^17,53^ However, instead of looking at cooperative coupling, excited state polaritons, or direct VSC with solvent, we mainly investigate the process of solvation under ground state VSC and weak excited state-cavity coupling. We employed pump–probe spectroscopy to study nonequilibrium dynamics of Re­(4,4′-bipyridine-2,2′-COOH)­(CO)_3_Cl complex (ReC0A) in an infrared microcavity. This molecule is a well-studied candidate catalyst for CO_2_ reduction technologies due to its ability to efficiently convert CO_2_ to CO,^54,55^ and in related Re­(I) tricarbonyl–diimine systems, mid-IR CO-stretch vibrational polaritons have been observed in etalon microcavities, underscoring the strong oscillator strength of this family.^56^ In an earlier transient spectroscopic study of ReC0A in dimethylformamide (DMF), visible pump excitation revealed ultrafast solvation-induced, time-dependent vibrational Stokes shifts, highlighting peak shift magnitudes and dynamics that are key to understanding solvent–solute interactions.^57^ Kubarych et al. also showed that preferential donor solvation (≈20% TEOA/THF) preassembles the Re–donor pair, enabling <100 ps electron transfer to the catalytically active Re and correlating with maximal CO output in CO_2_ reduction.^58^ Here, we investigate the spectroscopic signatures of the solvation dynamics and whether the solvent-induced dynamical peak shifts are modified under vibrational strong coupling.

Even though it seems nontrivial for VSC to have an effect on excited state dynamics, it can, in principle, back-act on the ensuing MLCT relaxation because the cavity and molecule ensemble redefine both the initial electronic state and the dynamical bath that the excited molecule feels. At thermal equilibrium, microcavity VSC has been hypothesized to modify electronic polarizations in individual molecules, such that photoexcitation begins from a shifted charge distribution and altered first-solvation shell, changing the initial conditions for charge localization and solvation dynamics.^59^ Beyond these local effects, the cavity mode itself may act as a structured, dissipative environment that reshapes the bath spectral density and correlation functions, with potential consequences for dynamical Stokes shift and vibrational relaxation rates.^12,16^ Collective light–matter coupling has also been proposed as a modifier of solvation and aggregation microenvironments, leading to changes in local polarizability and thermal fluctuations even in the absence of explicit polariton formation with an electronically excited state.^60^ Finally, recent theory suggests that the weakly coupled reservoir of dark molecular states acquires distinct delocalization and physicochemical properties within a cavity, implying that polariton participation is not strictly required for microcavity confinement to influence molecular function, including reactivity.^61–63^ Together these studies make a plausible case that ground-state VSC could potentially change the MLCT Stokes-shift dynamics.

In contrast to most prior studies on vibrational polariton nonequilibrium dynamics that have utilized two-dimensional (2D) IR^38,41,64–66^ and transient/2D visible (VIS) spectroscopies,^67,68^ we leverage transient visible pump and IR probe spectroscopy, a technique that has garnered interest but, to the best of our knowledge, has yet to be applied in VSC experiments. In our setup, the visible pump excites the ReC0A complex into an electronically excited state and the infrared probe detects the excited state optical response. This approach provides insight into ultrafast vibrational dynamics and the effects of VSC on electronically excited molecular systems in infrared microcavities. Our main results are the experimentally observed new signatures of time- and angle-resolved solute–solvent dynamics in an infrared microcavity, while presenting a new strategy for characterizing nonequilibrium vibrational dynamics under VSC.

## Results and Discussion

### Solvation Induced Stokes Shift of ReC0A-DMF: Outside Cavity

We first study the solvation-induced dynamical Stokes shift of ReC0A in DMF outside a Fabry–Pérot microcavity by visible-pump/infrared-probe transient absorption spectroscopy, using a 31 μm path length flow cell and a ReC0A concentration of approximately 25 mM. The ReC0A complex consists of a rhenium­(I) metal center coordinated by a bipyridine-COOH (bpy-COOH) ligand and three carbonyl (CO) ligands, along with a chloride (Cl) ligand. The molecular structure is shown in the inset of Figure 1a. The UV/vis absorption spectrum (Figure 1a) shows the singlet metal to ligand charge transfer (^1^MLCT) band centered at ∼400 nm. The FTIR spectra of The ReC0A complex in DMF, shown in Figure 1b, exhibits three CO stretch modes at 2020, 1919, and 1900 cm^–1^ corresponding to the in-phase symmetric (A′(1)), antisymmetric (A″) and the out-of-phase symmetric stretch (A′(2)), respectively.^54^

**Figure 1:**
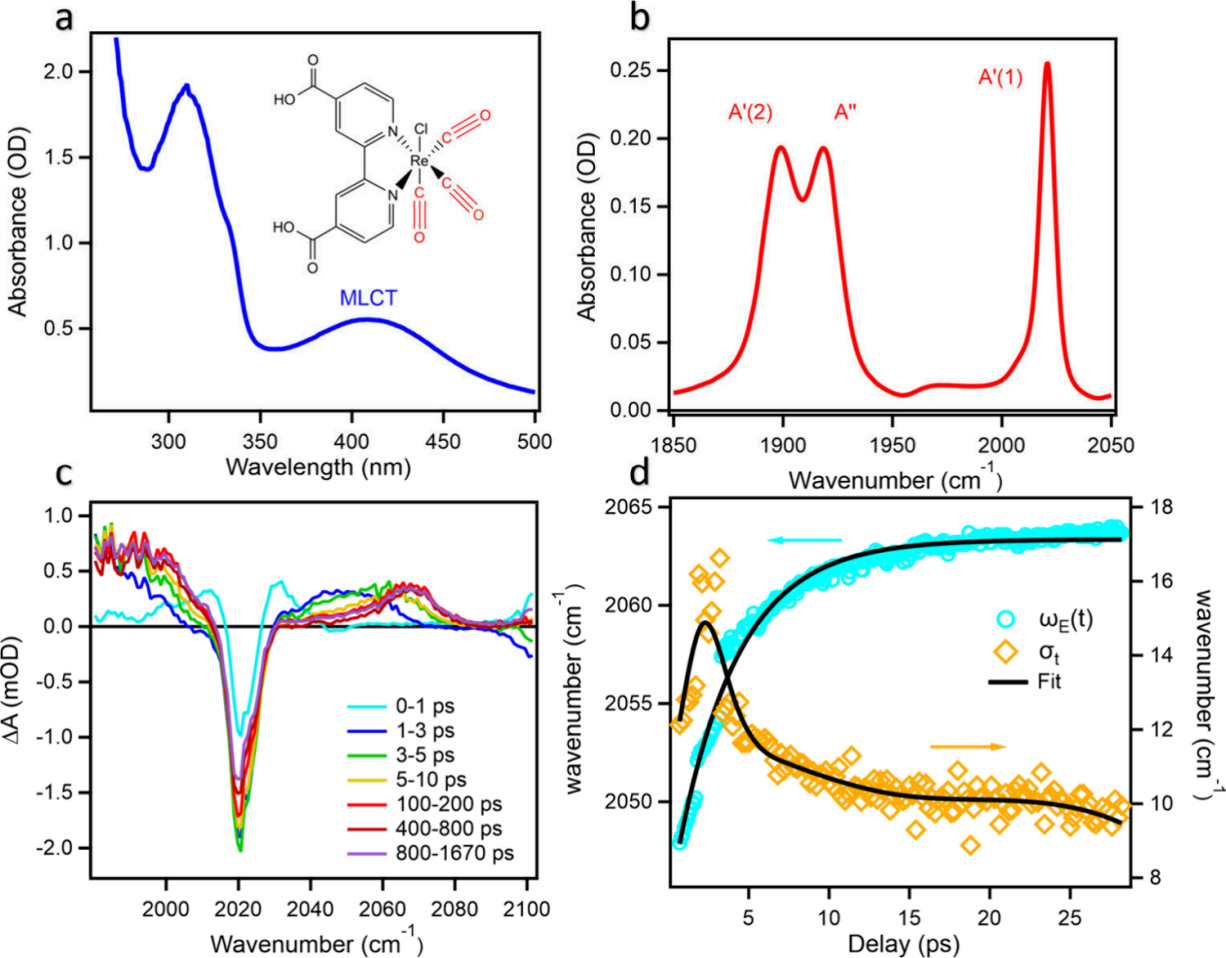
Transient measurements of ReC0A outside cavity. (a) UV–vis spectrum of the ReC0A complex. Inset: ReC0A complex molecular structure. (b) FTIR spectrum of ReC0A focusing on CO stretching mode. (c) Transient IR absorption spectra measured with a 400 nm pulse excitation and broadband IR pulse probe. The excited state absorption feature located at 2040 cm^–1^ at early times undergoes a blue shift toward higher frequencies (∼2070 cm^–1^) during the first 10–20 ps. (d) Temporal evolution of the excited peak position ω_abs_ (cyan sphere) and width σ_t_ (orange square), obtained from fitting the transient spectra in (c).

The transient IR absorption difference spectra measured with 400 nm excitation (Figure 1c) of the A′(1) mode shows a ground state bleach (GSB) of the 2020 cm^–1^ mode and its time-dependent positive excited state absorption (ESA) peak of the higher frequency CO stretching mode (Figure 1b). The ESA initially grows in at ∼2040 cm^–1^ and gradually blue-shifts toward higher frequency over a few ps, reaching its final position at ∼2070 cm^–1^, after which the ESA peak intensity decays slowly on the nanosecond scale. The spectral response of the lower frequency modes falls mostly out of the measured spectral window and is not discussed here. These results agree with those previously obtained and are well understood.^57^ The transient absorption spectrum at each delay time can be fit by a sum of GSB and ESA signals, each described by a Gaussian function. The GSB peak position and width are fixed to the values obtained from the FTIR spectrum, while the center and width of the ESA peak are fitting parameters. The details of the fitting process are described in the Supporting Information (SI.4). The position and width of the ESA peak extracted from the fit are plotted as a function time in Figure 1d. The peak blue-shifts within the first ∼10 ps and starts to reach a steady state position at longer time. This time-dependent peak shift is attributed to the solvation dynamics and can be fit to eq 1.

1 $$\overset{®}{\omega_{E}\left(t\right)\;}=\omega_{E,0}+\left(\omega_{E,\infty }-\omega_{E,0}\right)\left[1-e^{-t/\tau }\right]$$

In eq 1, *t* is the pump–probe time delay, τ is the solvation time, and *ω*_*E*__,0_ and *ω*_*E*__,∞_ are the center of ESA peak at *t* = 0 and at *t* → ∞ (Figure 1d, SI.4). The global fitting result gives *ω*_*E*__,∞_ ∼ 2063.4 cm^–1^, *ω*_*E*__,0_ ∼ 2044.7 cm^–1^ and τ ∼ 3.77 ps. The ESA peak width initially increases up to ∼2 ps before decreasing. The decrease is consistent with vibrational cooling as energy dissipates into lower-frequency vibrational modes. The reason for the initial increase is not entirely clear, but at early times, the excited state peak overlaps with the ground state bleach, which may affect reliable fitting.

The mechanism of the ESA shift has been investigated in a previous work.^57^ The 400 nm excitation of the ^1^MLCT excited state transfers an electron from the d-orbitals of Re­(I) to the π* orbital of bipyridine, creating a dipole moment change in the molecule.^54^ This shift in the solute dipole moment drives the surrounding solvent molecules into a nonequilibrium configuration, inducing an immediate polarization response and generating a time-dependent reaction field. As solvent molecules reorient to stabilize the excited state, the local electrostatic field around the solute evolves, modifying its vibrational frequencies and resulting in a time-dependent Stokes shift. Beyond dielectric solvation, prior studies show that the early IR shift also reflects sub-ps ^1^MLCT to ^3^MLCT state conversion^69,70^ and subsequent equilibration within the triplet manifold, evolving charge localization/structure^71,72^ and vibrational cooling;^70^ transient 2D-IR directly correlates ground- and excited-state CO modes via cross-peaks.^73^ We do not believe relaxation within the electronic triplet manifold materially affects the dynamics we analyze, as this redistribution occurs on sub-200 fs time scales in related transition-metal complexes; our fits begin after this time window.^74–76^ Both the vibrational relaxation and solvation dynamics produce the observed excited state absorption (ESA) blue-shift and peak width change, a manifestation of the evolving solute–solvent interaction as the system approaches equilibrium in the electronically excited state. In addition, the initial rapid shift corresponds to the inertial response of the solvent molecules, while the slower component is associated with their diffusive reorientation and the corresponding electronic reorganization of the solute density.

### ReC0A-DMF inside the Cavity

To investigate the effect of VSC on solvation dynamics, we prepare a sample of a 23 mM solution of ReC0A in DMF solution within a microcavity comprising two high-reflective dielectric mirrors separated by two stacked spacers with a total thickness of 31 μm (Figure 2a). The details of the sample preparation are provided in the Supporting Information (SI.1). The dielectric mirrors are designed to have a high transmission at around 400 nm, allowing us to excite the sample at the ^1^MLCT transition around 400 nm while probing the vibrational polariton. The transmission spectra of the empty cavity, Figure 2b inset, show a series of evenly spaced cavity modes arising from the Fabry-Pérot resonance condition. By varying the incidence angle of the probe beam (see Supporting Information, SI.3), the microcavity resonances can be tuned across the vibrational transitions.^35^ In this work, we focused on the 2020 cm^–1^ CO stretching mode, which has sufficiently strong absorption and narrow enough line width for strong coupling to the microcavity to occur. Figure 2b shows the FTIR spectrum of ReC0A in DMF (black dashed line) overlaid with the cavity transmission spectrum at an incident angle of ∼9.9° (blue solid line). At this condition, the cavity mode is resonant with a vibrational transition (blue) and two polaritonic states emerge, with equal photonic and vibrational character. We observe upper polariton (UP) and lower polariton (LP) responses, shown in red dots, centered around 2011 cm^–1^ and 2028 cm^–1^, respectively. The frequency splitting Ω_R Ω_*R*_ between the UP and LP at resonance, i.e. the Rabi splitting, is determined to be ∼18 cm^–1^. The splitting satisfies the strong coupling criteria, i.e., it is larger than the full width at half-maximum (fwhm) of both molecular absorption (∼6.9 cm^–1^) and cavity transmission (∼7.8 cm^–1^). One significant observation from our experiments is the discrepancy in the observed Rabi splitting between the FTIR and transient IR setups. This discrepancy is attributed to spatial variation in cavity length in our sample. Different Rabi splitting values were observed due to the much larger beam sizes used in the FTIR measurement, which samples a broader distribution of cavity lengths. As described in Supporting Information SI 5.1, this discrepancy can be accounted for by assuming a cavity length distribution width of approximately 50 nm. This discrepancy and its magnitude could be reproduced across various measurements. For simulations and analyses of the transient absorption data, we used the Rabi splitting measured in the transient IR setup. We operate the cavity in the vibrational strong-coupling regime for the CO v = 0→1 transition, as verified by angle-resolved linear transmission showing UP-LP anticrossing. VSC here denotes the existence of hybrid light–matter eigenmodes and does not depend on their population. Figure 2c presents the simulated transmission dispersion of the ReC0A-filled microcavity. Near the resonance condition at ∼9.9°, it shows both the UP and LP bands with a Rabi splitting. At higher and lower incident angles, the Fabry-Pérot cavity is no longer in resonance with the molecular vibration, and only the cavity transmission is observed. At incident angles of 18–20°, the transmission of a lower order cavity mode appears at a lower wavenumber region. The angle dependence shown here is relevant for later transient IR experiments, where the probe angle is adjusted to access different spectral regions. For example, by tuning to a larger angle, the cavity transmission modes appear in the 2040–2060 cm^–1^ region, allowing the probe of excited-state absorption bands.

**Figure 2:**
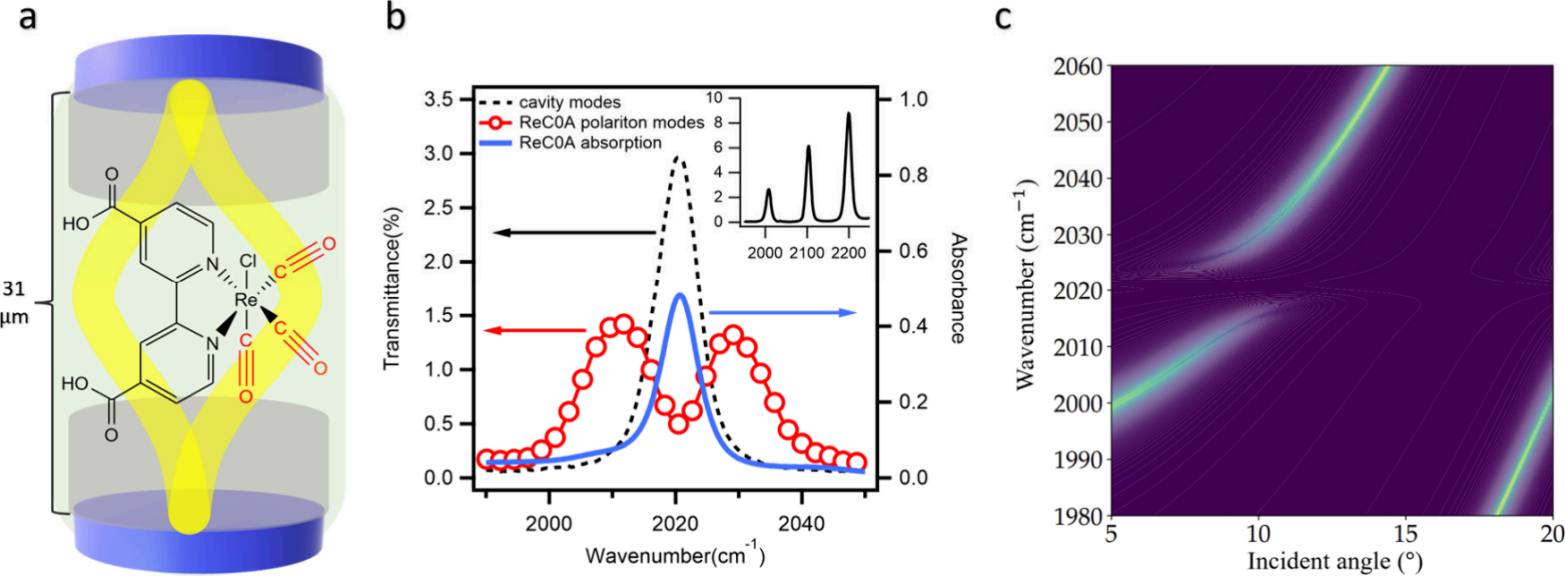
Microcavity schematic representation and frequency-domain spectra and dispersion. (a) Schematic representation of Fabry-Pérot cavity consisted of two dielectric mirrors separated by 31 μm space filled with ReC0A solution in DMF. The photon mode (yellow line) is for illustration only; the actual mode coupled is 35. (b) Fourier transform infrared (FTIR) absorption spectrum of the ReC0A (blue solid line, right axis), transmission spectrum of the bare Fabry–Pérot cavity filled with neat DMF (dashed black line, left axis), and transmission spectrum of the resulting polariton branches under strong coupling between ReC0A and the cavity mode (red open circles, left axis). Inset: empty cavity transmission. (c) 2D contour plot of simulated transmission spectrum of infrared microcavity filled with ReC0A-DMF sample as a function of probe wavelength and the incident angle of the probe beam relative to the cavity axis.

### Solvation Induced Stokes Shift of ReC0A-DMF in IR Microcavity

We performed initial transient IR transmission spectroscopic measurements at the incidence angle, where light and matter are resonant. For all the intracavity spectra, we plot normalized differential transmission defined as ΔT = [I_on_ – I_off_]/I_0_, where I_0_ is the baseline of the incident IR light. The transmission spectrum of the sample before excitation, as measured by the femtosecond IR setup, is shown in Figure 3a. We refer to this spectrum as ‘on-resonance’ because the probed cavity mode is tuned to (i.e., spectrally overlaps) the ground-state CO ν = 0→1 transition. It shows the expected upper and lower polariton peaks at 2012 cm^–1^ and 2026.5 cm^–1^ respectively, with a Rabi splitting of 14.5 cm^–1^ (see SI 5.1). The transient IR transmission difference spectra measured with 400 nm excitation at selected delay times are shown in Figure 3b and the full set of time-resolved spectra spanning 1 to 350 ps can be found in Supplementary Figure S4. The transient spectra are characterized by two prominent derivative features around the upper and lower polariton modes. The 400 nm pump pulse excites ReC0A to the ^1^MLCT excited state, which depletes the ground state population and consequently reduces Rabi splitting. This effect, known as Rabi splitting contraction,^77^ causes the two polariton peaks to shift toward the bare molecular frequency, giving rise to the derivative-like features in the transient difference transmission spectra. It is important to note that Rabi splitting contraction is determined by the decrease of ground state population, which does not change in the 1 ps to 10s of ps time window that will be discussed below. Thus, all spectral evolutions in this time scale are caused by the change in excited state vibrational spectra, reflecting the excited state dynamics.

**Figure 3:**
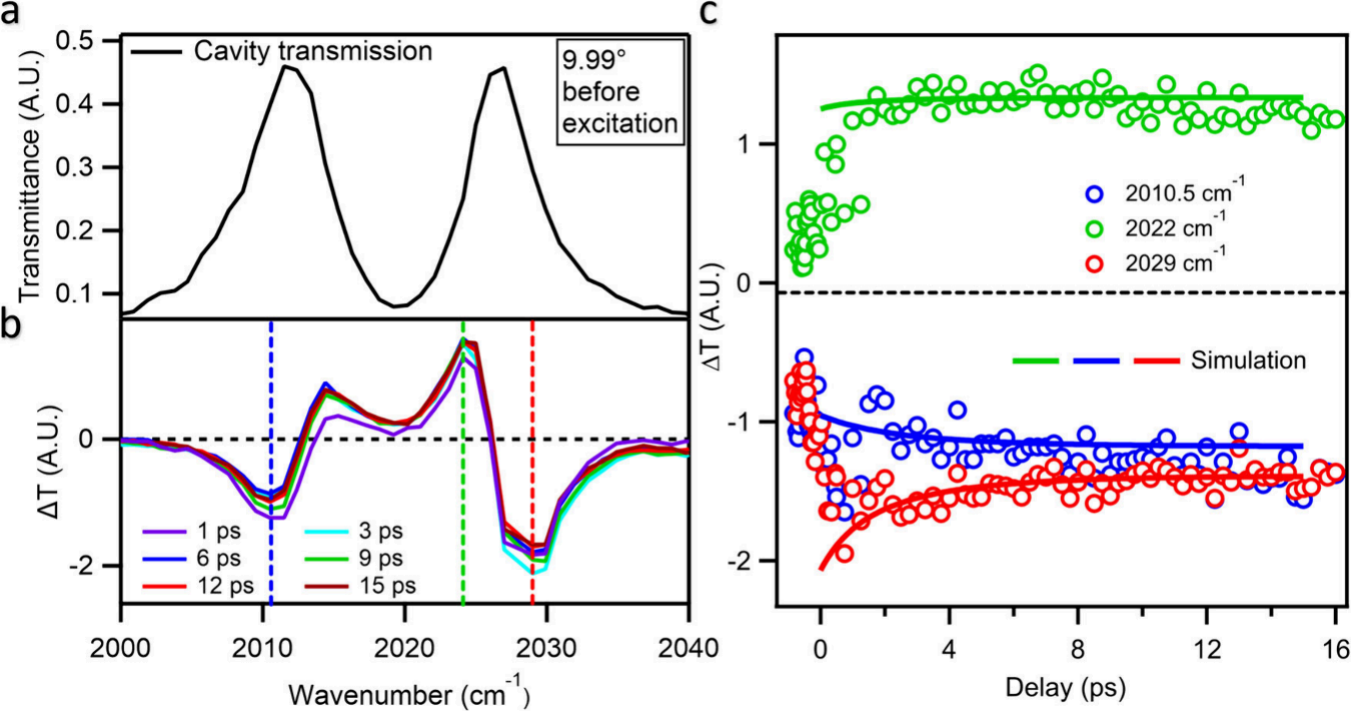
Measured on-resonance polariton dynamics for ReC0A within the microcavity. “On-resonance” denotes that the probed cavity mode is tuned to the ground-state CO ν = 0→1 transition. (a) Transmission spectrum of on-resonance coupled ReC0A before visible excitation, showing clear upper polariton (UP) and lower polariton (LP) features. (b) Transient difference transmission spectra of on-resonance coupled ReC0A indicate delay times after 400 nm excitation. The incidence angle of the probe was chosen to tune the microcavity mode to be nearly resonant with the CO stretch at 2020 cm^–1^. The derivative line shape around the UP and LP modes are induced by Rabi splitting contractions caused by photoinduced depletion of ground state molecules. Stronger features are observed near the UP frequency, attributed to overlap with the electronically excited-state absorption band, which weakly couples to the microcavity. (c) Kinetics at three different frequencies (blue, green, and red vertical dashed lines) of the spectra and their fit according to a model described in the main text.

A clear asymmetry is observed between the LP and UP derivative features, with a larger depletion in transmitted light near the UP relative to the LP, which is attributed to the effect of the absorption of the electronically excited state population. The electronically excited ReC0A exhibits a time-dependent blue-shifted vibrational mode at around 2040–2060 cm^–1^, as shown in Figure 1c. Under our experimental conditions, only a small fraction of ReC0A molecules are electronically excited by the pump pulse. When the cavity is resonant with the ground state absorption at 2020 cm^–1^, the ESA is expected to weakly couple to the tail of the cavity mode. This weak coupling leads to a significant reduction in transmitted light near the UP frequency as the molecular ESA band partly overlaps with the UP. This overlap explains the stronger transient features near the UP frequency, contributing to the observed asymmetry in the transient transmission difference spectra. Three kinetic cuts are extracted and plotted in Figure 3c, corresponding to the key features of the transient transmission spectra. The kinetics at 2010.5 cm^–1^ (blue), 2022 cm^–1^ (green) and 2029 cm^–1^ (red) show the formation of these features on the <1 ps time scale followed by smaller changes in their amplitude on the few ps time scale. The latter corresponds to the blue-shifting of the ESA band, which evolves as the system relaxes. These features are consistent with the time scale of the ESA blue shift observed in the transient spectra of the molecule outside cavity (Figure 1c,d).

### Excited State Evolution Monitoring via Transient Angle-Resolved Dynamics

To monitor the solvation-induced dynamical Stokes shift of the excited molecules inside the microcavity, we measure the transient transmission spectra at incidence angles where microcavity modes spectrally overlap with the ESA band, which we denote as “off resonance”. Note that strong coupling of the ground-state CO is verified by the observed Rabi splitting, whereas for the ESA the much smaller excited-state population, together with intentional detuning at our probe angles, places it in the weak-coupling regime (no excited state Rabi splitting is observed). We want to emphasize that “VSC” refers exclusively to the strong coupling of electronical ground state CO *v =* 0 to 1 transition to the cavity vacuum field. The coupling and formation of polaritons persist irrespective of whether there is a probe and its angle; changing the detuning does not switch VSC on or off. By contrast, the MLCT ESA is off-resonant and involves only a small excited-state fraction; therefore, its collective coupling is small, and no electronically excited state polariton forms, and the ESA serves as a linear reporter of the solvation dynamics. Importantly, VSC remains present throughout, and the probe does not switch VSC on or off. This enables us to track the solvation nonequilibrium response via weak coupling of the ESA and resonant microcavity modes. The microcavity transmission as a function of incidence angle is given in the Supporting Information (SI.3). Note that a fundamental difference between the outside and inside cavity measurements is that the microcavity measurement allows probing only within the cavity mode transmission window. Therefore, in measurements with the incidence angle fixed at a microcavity mode in resonance with the relevant ground-state vibrational transition (2020 cm^–1^), almost no light is transmitted at the excited-state absorption window (2040–2070 cm^–1^), and as the ESA peak blue shifts, it moves outside the observation window. This issue can be resolved by probing the system at variable incidence angles. When the probe is tuned on resonance with UP LP, it excites those pre-existing eigenmodes; when it is significantly detuned (as in our transient ESA windows at 13.1° and 16°), it predominantly excites matter-like transitions with negligible polariton admixture. At sufficiently high incidence angles, significant overlaps occur between the microcavity mode and the time-dependent ESA, resulting in weak coupling that can be used to track the kinetics.

Because the microcavity acts as a dispersive transfer function which includes filtering, polariton dispersion, and Rabi-contraction as well as derivative line shapes, the apparent peak motion inside the cavity is not simply related to the ESA center frequency as it is outside the cavity. Direct exponential fits of the intracavity kinetics would therefore not yield a reliable solvation rate. Instead, we adopt a forward-model comparison: we take the bulk-extracted Stokes-shift parameters, and introduce them as input into the cavity response model (SI 5.1) to generate simulated intracavity traces at the same probe wavenumbers. Because the MLCT ESA is weakly coupled, we believe that this treatment is appropriate. Agreement between the simulated and measured intracavity kinetics constitutes our quantitative comparison.

At an incident angle of 13.1°, the cavity mode is centered around 2048 cm^–1^ with a transmission window of 2030–2060 cm^–1^ (Figure 4a). The transient transmission difference spectra, shown in Figure 4b, exhibit a derivative feature, with an increased transmission at the lower energy side (∼2030–2047 cm^–1^) and decreased transmission at the higher energy side (2047–2060 cm^–1^) of the cavity transmission window. The derivative feature undergoes a blue shift, with the positive transmission increasing, and the negative transmission decreasing within the first 10 ps before reaching a steady-state feature. The decreased transmission on the high-frequency side is attributed to the increased absorption of excited molecules in this spectral region. As the ESA peak shifts to higher energy due to solvation dynamics over a few picoseconds, the amplitude of the decreased transmission is expected to diminish gradually as the peak moves outside the transmission window.

**Figure 4:**
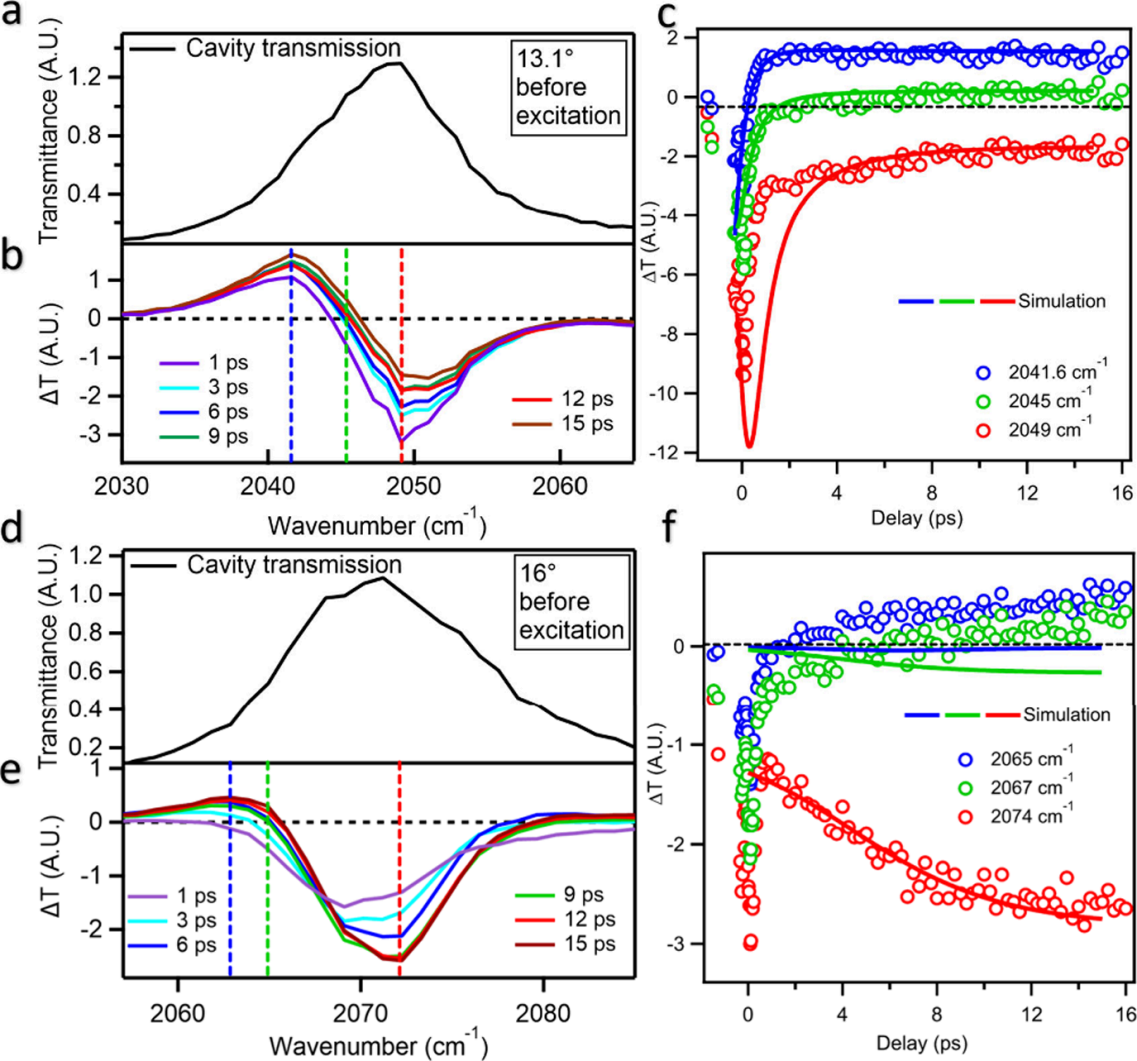
Measured off-resonance dynamics for ReC0A within the microcavity. “Off-resonance” denotes detuning from the CO ν = 0→1 transition; the cavity mode only overlaps the MLCT ESA band to read out the Stokes shift (ESA remains weakly coupled). (a,d) Transmission spectrum before excitation, (b,e) transient difference transmission spectra at indicated delay time after 400 nm excitation and (c,f) time-dependent transmittance change (kinetics) at indicated IR frequencies of ReC0A inside cavity at a probe angle of 13.1° (a,b,c) and 16° (d,e,f). The selected probe IR frequencies in (c) and (f) are also indicated in (b) and (e), respectively.

At an incident angle of 16°, the cavity transmission window is centered at ∼2071 cm^–1^ (Figure 4d). When tuned on resonance, the probe excites pre-existing UP/LP eigenmodes; when significantly detuned (as here), it predominantly excites matter-like ESA transitions. The transient difference transmission spectra (Figure 4e) also exhibit a derivative feature, with increased transmission at the low frequency side and decreased transmission on the higher-energy side, although the latter feature has much higher amplitude. Unlike the measurement in the lower frequency window, the amplitude of the negative transmission change feature increases with time, reflecting the blue-shifting of the excited absorption into this spectral window over a few picoseconds.

To further understand and validate the experimental results, we simulated the pump–probe transmission spectrum of ReC0A in an IR microcavity by considering the Fabry–Perot transmission properties in the presence of a nonlinear dielectric medium with inhomogeneous absorption broadening.^78^ In our model, we assume the molecular dynamics remains unchanged inside and outside the microcavity. Under this assumption, if the model successfully explains most of the experimental data, it suggests that the excited-state Stokes shift rate constant is negligibly affected by VSC. Conversely, any significant deviations would imply that the rate constant is modified under VSC (see Supporting Information, SI.5).

The on-resonance simulated spectra without and with the inclusion of the ReC0A ESA band are shown in Figure 5a and 5b. The simulation assumes an incoherent molecular electronically excited-state population of 1%, following the same dynamics as the molecules outside a microcavity. After visible excitation, the derivative features of the polaritons are observed in both cases, confirming their origins indeed from Rabi splitting contraction. When the ESA is not included in the simulation in Figure 5a, the depletions near the UP and LP are fairly symmetric and do not evolve over time. However, when the time-dependent ESA evolution is included, the simulated spectra display an asymmetry similar to the experiments, with the depletion near the UP being greater than that near the LP. This agrees well with the experimental data. Additionally, with the ESA considered, the transmitted light intensity near the UP frequency gradually increases with time (see insets of Figure 5a, 5b), consistent with experimental results (Figure 3b) and supporting the interpretation that this behavior arises from the weak coupling between the ESA band and the tail of the cavity mode.

**Figure 5:**
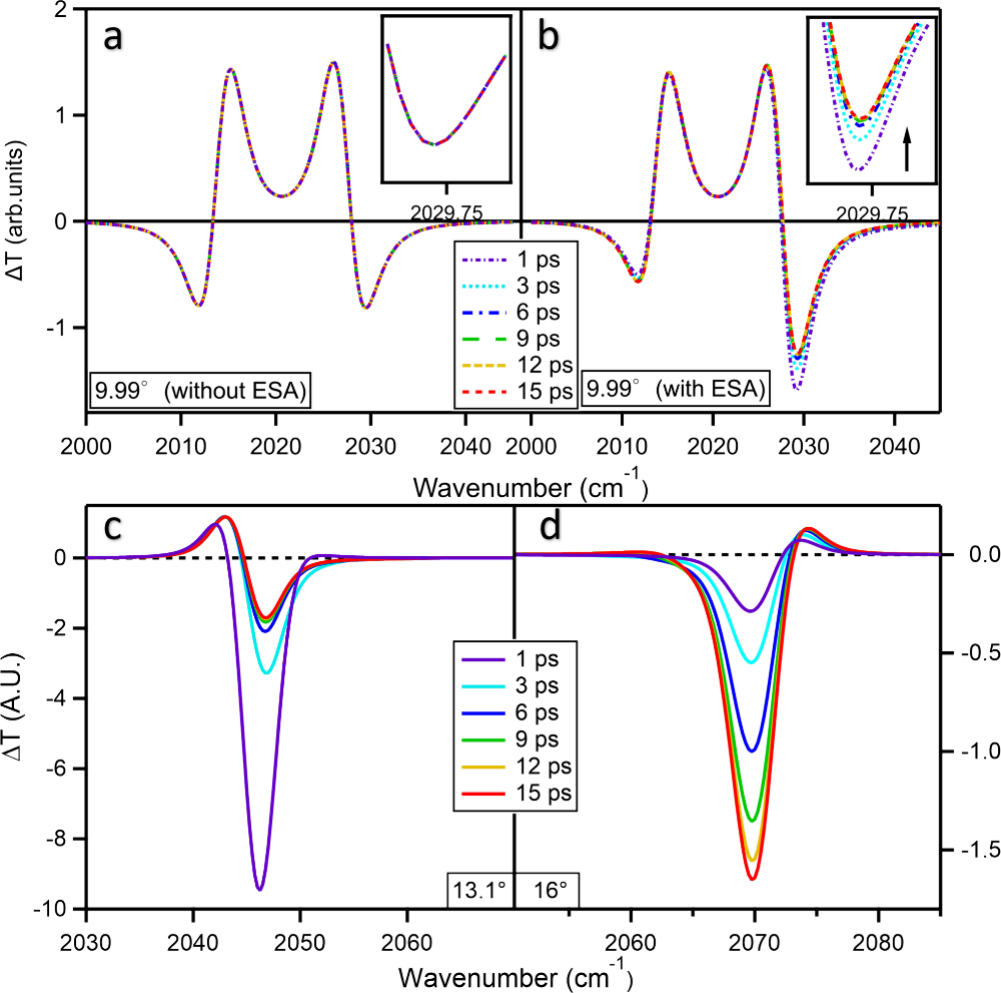
Simulated Transient Spectra of ReC0A inside the microcavity. (a) On-resonance simulated transient spectra without ESA contributions. The depletion features near UP and LP are symmetric and remain static over time, indicating that ESA effects are responsible for the observed spectral asymmetry. Inset: zoomed in window for the 2030 cm^–1^ depletion. (b) On-resonance simulated transient spectra include ESA effects, assuming an incoherent molecular electronically excited-state population of 1%. The asymmetry in the transient response, with stronger depletion near the upper polariton (UP) compared with the lower polariton (LP), agrees well with experimental results. Inset: zoomed in window for the 2030 cm^–1^ depletion. (c) Simulated off-resonance transient spectra at an incident angle of 13.1°, corresponding to the experimental spectra in Figure 4b. (d) Simulated off-resonance transient spectra at an incident angle of 16°, corresponding to the experimental spectra in Figure 4e.

The simulated off-resonance spectra are shown in Figure 5c and 5d, corresponding to the experimental results shown in Figure 4b and 4e. These simulated spectra features are in qualitative agreement with experimental results. While the bleach signal, representing the absorptive feature due to weak ESA coupling to the overlapping microcavity mode, aligns well with experiments, the simulated spectra at 13.1° (centered around 2047 cm^–1^) show a significantly stronger early time bleach feature in transmission compared to experimental observations. We believe that the discrepancy between theory and experiment at early times likely arises from the tails of the excited state peak. While the Gaussian model provides a better approximation of the line shape at later times, the early time ESA band is broadened primarily by anharmonic coupling between hot vibrational modes, which cannot be accounted for by the Gaussian line shape. In the simulation, the idealized line shape exaggerates the transmission changes in this spectral region. Furthermore, although the simulation correctly captures the positive peak, the relative amplitude and its ratio to the bleach are not in quantitative agreement with experimental data. This discrepancy is attributed to the experimental signal being heavily influenced by the evolution of the ESA line shape tails. The simulations employ a time-dependent line shape model of the bare dynamics that captures only the dominant features of the line shape and neglects effects such as DMF weak coupling and nonlinear solvent response, as the solvent is treated as a static dielectric continuum. However, because our study focuses on the time-dependent Stokes shift evolution rather than the absolute early time intensity, we think these deviations do not affect the interpretation of solvation dynamics under a VSC.

The transient measurements at 16° also display an unintuitive positive peak feature on the low frequency side, particularly in the lower-frequency window that is not well captured by the simulated. We attribute this to the broadening of microcavity mode caused by interactions between the excited molecules, solvents, and the microcavity itself (see Supporting Information). Following visible excitation, interactions between light, mirrors, molecules, and solvent molecules induce complex refractive index changes in both the sample and the mirrors. As a result, the absolute transmission of the targeted microcavity mode broadens. When comparing the transmission before and after excitation, a derivative-like feature emerges, as shown in Figure S6.

To test whether the Stokes-shift rate outside the cavity reproduces the intracavity data and thus whether the excited-state rate is altered under VSC, we compare kinetic cuts from the simulations with those measured experimentally. The simulated cuts are shown in solid line in Figures 3c, 4c and 4f. To ensure a meaningful comparison, the kinetic traces in simulations and experiments were selected to correspond to the key spectral features, even though their exact frequencies do not match perfectly match. In the simulated spectra, as the incident angle increases and the effect of the inhomogeneity of the cavity lengths becomes more pronounced, it becomes increasingly difficult to precisely match the cavity mode parameters. To account for this, we selected kinetic traces in the simulation at frequencies that best align with the characteristic peak maxima or bleach minima rather than strictly matching the experimental frequencies. This approach ensures that the qualitative evolution of key spectral features is meaningfully compared. As shown in Figure 3c, a single global rescaling of the simulated kinetics at 2022 and 2029 cm^–1^ traces align them well with the measured one. For the on-resonance case, the measured kinetics at 2010.5 cm^–1^ include an excited-state absorption (ESA) contribution of the lower frequency CO modes on the red side of the 2020 cm^–1^ transition (Figure 1C). This contribution is not taken into account in the simulations. We applied an additional negative offset to the 2010.5 cm^–1^ kinetics to account for the missing ESA and allow all three to be compared on the same vertical scale. Overall, the simulated kinetics capture well the excited state Stokes shift dynamics inside the microcavity. In Figure 3c, the kinetics at 2029 cm^–1^ show a time-dependent increase in transmission due to the appearance of the blue-shifted ESA around 2040 cm^–1^. As observed in Figure 1c, the shift is rapid within the first few picoseconds, with the ESA quickly moving out of the probe window defined by the cavity mode line width. When the cavity mode is at a higher frequency, probing the end of the ESA shift, the kinetics trace shows a gradual decrease in amplitude, indicating that the ESA band is approaching the cavity mode as a function of time.

For the spectra measured at 13.1°, the selected kinetics at 2041 and 2045 cm^–1^ are also well described by the simulated results (Figure 4c). However, the kinetics at 2049 cm^–1^ do not show this gradual decrease. Instead, the negative peak appears almost instantaneously and decreases monotonically with time. For the measured kinetics at 2049 cm^–1^, the center of the simulated microcavity mode was selected to be 2046 cm^–1^ with a 2 cm^–1^ difference that is within the experimental calibration uncertainties, to avoid the strong bleach signal that appears in the simulation. As discussed above, the discrepancy is likely caused by the inadequate description of the line shape of vibrationally hot molecules by the Gaussian function used in our modeling. For the spectra measured at 16°, the selected kinetics at 2074 cm^–1^ is also well described by the simulated results (Figure 4f). The kinetics at 2065 cm^–1^ and 2067 cm^–1^, corresponding to the positive features at the lower frequency side of this spectral window, are not well described by the simulated results, which is attributed to inadequate description of the positive peak at the lower frequency side, as described above. Importantly, the overall good qualitative agreement between simulation and experiment suggests that there is no significant modification to the underlying excited state solvation dynamics of the ReC0A complex under vibrational strong coupling.

To fully understand the dynamics of ReC0A in DMF within the cavity and explore additional solvent and cavity effects, we examined the perturbed free induction decay (PFID) signal at negative time delays between the pump and probe. It seems the signal inside the cavity exhibits fast oscillations dominated by cavity-mediated field dynamics (interference with the solvent and mirrors) and not simple molecular PFID. A full analysis and controls are provided in SI5.3 and SI.6 (including comparison to out-of-cavity PFID and discussion of the observed fast oscillation frequency).

## Conclusion

This study provides an in-depth examination of the Re­(bpy-COOH)­(CO)_3_Cl complex (ReC0A) in DMF within a Fabry–Perot cavity, focusing on the effects of vibrational strong coupling (VSC) on solvent-induced time-dependent Stokes shifts. To the best of our knowledge, this is the first instance of transient visible pump IR probe studies in this field. By comparing the experimental and simulated results, we could interpret the derivative transient spectra within the cavity, accounting for weak and strong coupling effects. We also introduced a new methodology for the vibrationally excited state dynamics and spectral evolution inside cavity by employing angle-dependent probe measurement while carefully accounting for weak coupling effects. Our findings reveal that the electronically excited-state Stokes shifts inside the cavity largely align with those observed outside the cavity, suggesting that VSC does not significantly alter the nonequilibrium solvation dynamics of the ReC0A complex in our system. Under our coupling and resonance conditions, loss, and detuning we detect no effect; we therefore make no claim about other systems or regimes. We acknowledge that resonance and detuning can influence polaritonic observables,^79^ therefore whether VSC modulates excited-state solvation remains unestablished. In addition to the focus on Stokes shifts, we explored the role of cavity length fluctuations on the transmission spectrum and Rabi splitting frequencies and studied solvent-dominated PFID signals inside the cavity.

Future research could include enhancing the coupling strength, for example, by using a much higher Q-factor cavity with smaller effective volume, stronger field confinement, and smaller photon leakage rate, or using different coupling systems.^80–82^ Another approach could involve significantly increasing the excited state population to form excited state band polaritons. Such enhancements could potentially lead to more pronounced and interesting observations, providing deeper insights into the effects of the VSC on solvation dynamics and chemical reactivity. These variations provide stricter tests of whether the VSC can influence excited-state solvation/IVR; our present null result establishes the baseline and sensitivity. Moreover, the methodologies and insights from this study can be applied to other molecular systems and coupling scenarios, offering a versatile new framework for future exploration of polaritonic chemistry.

## Supplementary Material



## Data Availability

Additional experimental details, supplementary figures and data are available from the authors. Most data generated in this study are provided in the Supporting Information.

## Notes

Experimental details, cavity fabrication/characterization, angle-resolved linear spectra, simulation and fitting procedure, and discussion of PFID analysis and controls (PDF)
