# Supplementary material for: Excited State Dynamics of CO2 Reduction Catalyst under Vibrational Strong Coupling
Source: J Am Chem Soc. 2025 Oct 10;147(42):38320–30. doi: 10.1021/jacs.5c11060 (PMC12550848; doi:10.1021/jacs.5c11060)
Supplement: Supplementary file 1 [file ja5c11060_si_001.pdf]

Supplementary Information

Excited State Dynamics of CO_2 Reduction Catalyst under Vibrational Strong Coupling

Tao Jin[1], Sara T. Gebre[2], Christopher J. Miller[3], Clifford P. Kubiak[*3], Raphael F. Ribeiro[*2], Tianquan Lian[*2,4]

[1] Department of Physics, Emory University, 1515 Dickey Drive, Northeast, Atlanta, Georgia 30322, United States

[2] Department of Chemistry, Emory University, 1515 Dickey Drive, Northeast, Atlanta, Georgia 30322, United States

[3] Department of Chemistry and Biochemistry, University of California San Diego, 9289 S Scholars Dr, La Jolla, CA 92093, United States

[4] Department of Chemistry, University of Pennsylvania, 231 S 34th Street, Philadelphia, Pennsylvania 19104, United States

SI.1 Experimental Methods - Fabrication and Characterization of Optical Cavity

Dielectric mirrors were purchased from Universal Thin Film lab corp. The IR reflection and visible transmission spectra of the mirrors are shown in Fig.S1. The mirror is specifically designed for this project as it transmits 400 nm light (over 90% transmission) and has ~93% reflectivity around 5000 nm. Figure S1c shows the UV–vis "absorbance" near 400 nm measured with a grating spectrometer. The absolute OD values should not be interpreted as true absorption, because this measurement is sensitive to polarization and finite NA, and it uses an air reference/baseline; thus reflection/scatter losses are folded into the reported OD. We include this panel only as a sanity check that the mirror has sufficient transmission near 400 nm to enable our transient measurements. The optical cavity was prepared in a demountable IR cell (Harrick) comprising of two dielectric CaF_2 mirrors. Mirrors are separated by two PTFE Teflon spacers (25 and 6 μm) stacked on top of each other. The effective free spectral range of the cavity formed is about 86 μm and the mode order that will be used to be coupled to the sample is 35.

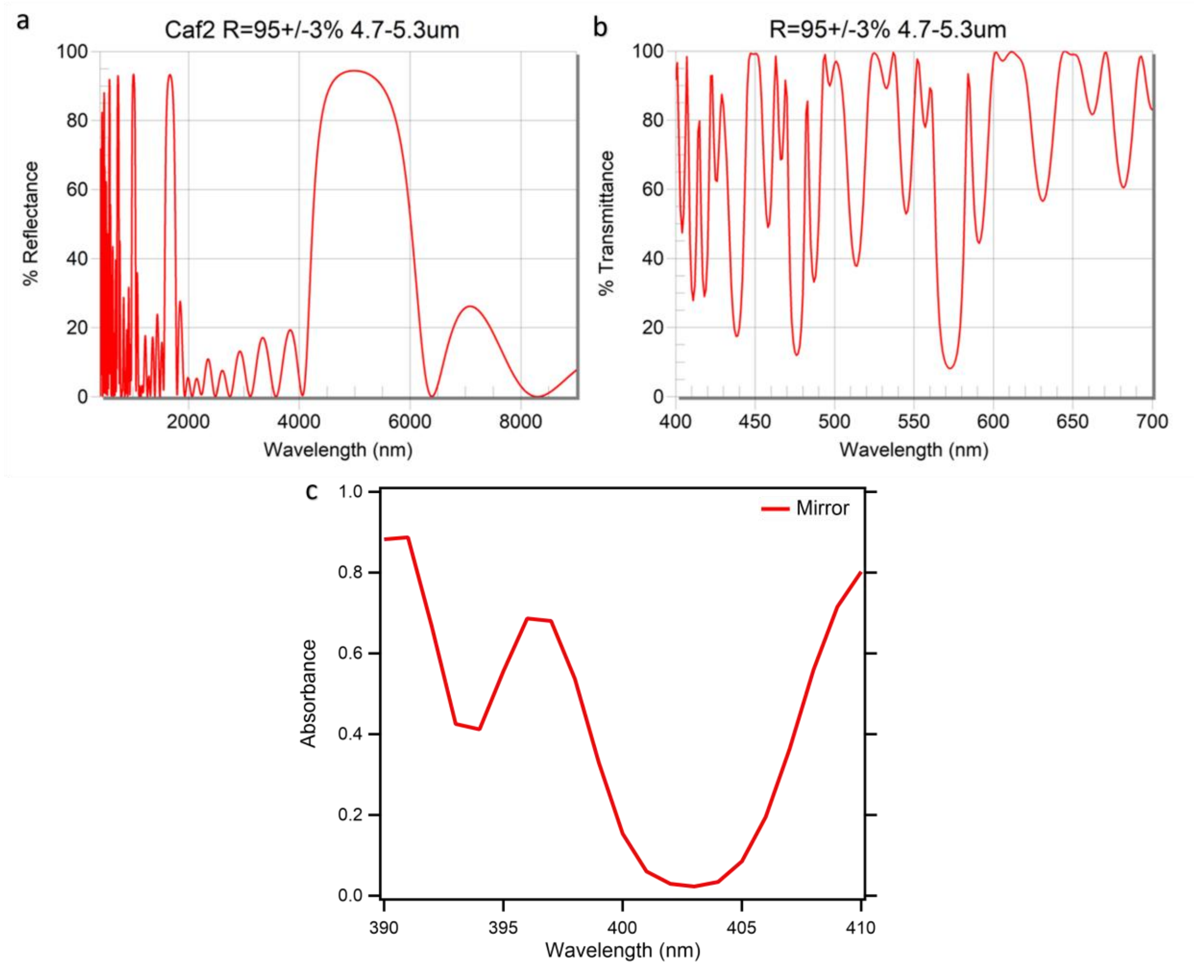

Figure S1. Reflectance (a), transmittance (b) and absorbance (c) Spectra of the dielectric mirrors used for the Fabry-Pérot cavity. The mirror is highly reflective around 5000 nm to cover CO stretch absorption and transmissive at visible lights for visible pump sufficiently interact with the sample.

SI.2 Transient Absorption Spectroscopy Setup

Femtosecond transient absorption experiments were conducted based on a regenerative amplified Ti:Sapphire laser system (Coherent Astrella, 1kHz repetition rate, 35 fs pulse duration and 5.5 mJ/pulse) to generate a fundamental 800 nm pulse. The fundamental pulse was split into two beams by a 50:50 beam splitter into the visible and IR optical parametric amplifiers (OPAs) (Light conversion). A 400 nm pump pulse was generated in the visible OPA through second harmonic generation in a type I BBO crystal. The IR OPA was used to generate a broadband IR probe centered around 4920 nm via difference frequency generation (DFG). A

chopper modulated the pump pulse by 500 Hz to obtain the pumped and unpumped absorption signal from the sample. A delay stage was set in the optical path of the pump pulse to provide time delay between pump and probe. A visible half wave plate was applied to adjust the polarizations between pump and probe to be at the magic angle. An IR half wave plate and polarizer pair was used to attenuate the probe without changing its polarization. The diameter of the pump and probe beam at the sample were around 700 μm and 500 μm, respectively. Data was collected on a nitrogen cooled mercury-cadmium-telluride (MCT) detector (PhaseTech) and analyzed in the QuickControl software from PhaseTech, Inc. The detector has 128x128 pixels across slightly over 100 cm^{-1} regions with the grating used, having around 0.85 cm^{-1} resolution. The Instrumental response function (IRF) was fitted by a Gaussian function with 190 fs full width half maximum. All experiments were performed at room temperature. While the cavity mirrors near 400 nm (Figure S1) can spectrally or temporally reshape the pump, our measured pump–probe IRF remains 100–200 fs across runs; any residual pre/post-pulse structure would chiefly perturb sub-IRF early-time amplitudes and does not affect the ps-scale ESA frequency dynamics used to extract the solvation time constant.

SI.3 Angle Tuning Experiment

Angle detuning experiments were conducted using a rotation stage from ThorLabs, with the cavity mounted on the stage. By rotating the cavity around its vertical axis, the effective cavity length is altered, which in turn shifts the cavity mode frequency. Figure S2 presents the simulated cavity transmission spectrum as a function of the incident angle. The complete simulated dispersion spectra as a function of incident angle of ReC0A-DMF is shown in Figure 2c. The frequency of the mode determines whether the coupling is targeted at the fundamental transition or the excited state absorption.

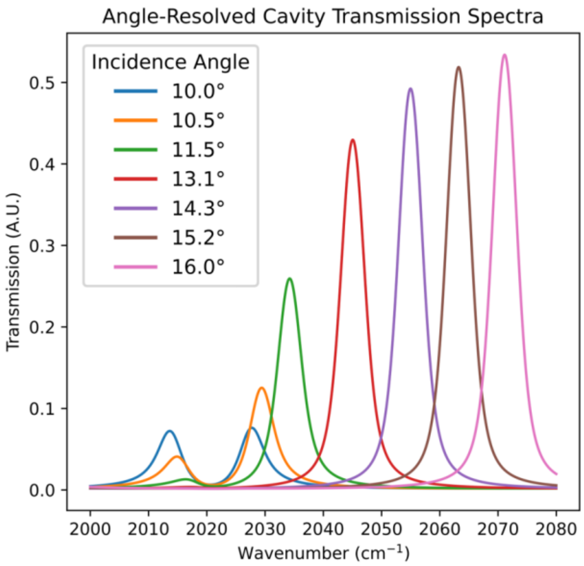

Figure S2. Simulated cavity transmission in different incident angles. The transmission shift is due to the effective cavity length realized. The cavity length and mode linewidth are tuned to match experiment ones. At >11° angle, the cavity mode is off resonance of the molecular fundamental transition but are expected to be coupled to ESA bands when ReC0A is excited. At around 10° angle, the coupling is present and LP and UP can be observed.

SI.4 Experimental Results

SI 4.1. Global Fitting of Excited State Stokes Shift Spectra

The ground state bleach (GSB) at 2020 cm^{-1} and excited state lineshape are globally fitted with a Lorentzian-Gaussian model:

$$f(x,t) = y_0 + Ae^{-(x-\omega_G)^2/2\sigma_G(t)^2} + \frac{B}{\sqrt{2\pi*\sigma_E{}^2(t)}}e^{-\left(x-\overline{\omega_E(t)}\right)^2/2\sigma_E{}^2(t)} \qquad (1)$$

where ω_G is the center position of GSB and $\overline{\omega_E(t)}$ of time dependent excited state absorption. The FWHM are calculated based on fitted σ_G and σ_E. Several experiments were conducted to confirm the reproducibility. Figure S3a shows the transient spectra at 1, 3 and 15 ps and their fit by the model described above. We extracted the parameters from the spectral fits and used them to generate kinetic traces, which we then compared to the experimentally measured kinetics at 2050 and 2070 cm^{-1} (Figure S3b). This comparison validates that our global fitting procedure accurately captures the temporal evolution of the signals. As expected, larger discrepancies appear at very early times: immediately after excitation the ESA band deviates from a Gaussian shape, but as the hot vibrational population cools the lineshape becomes increasingly Gaussian. Overall, the fitted and measured traces agree well, further justifying the

quality of our fits. The shaded area represents error bars of one standard deviation of the residual from the fit.

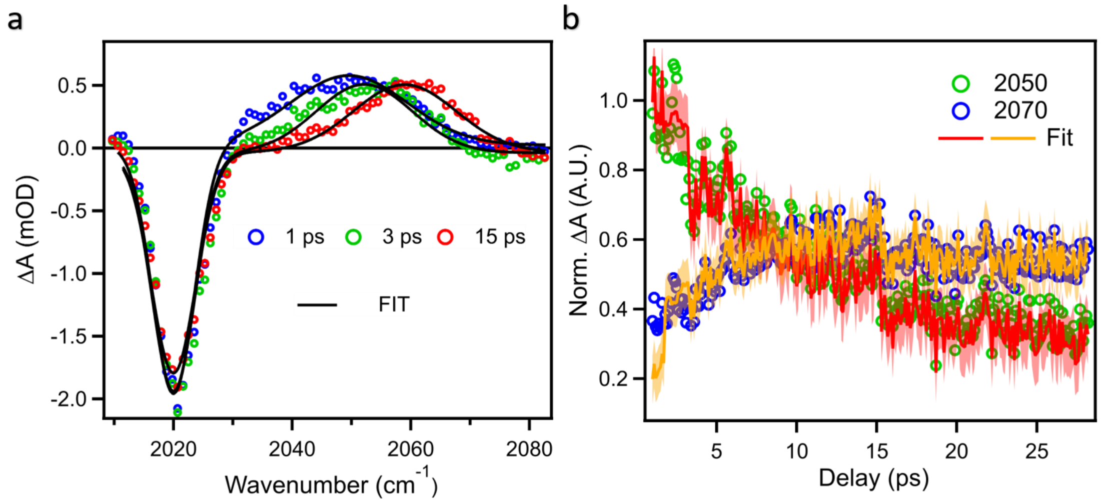

Figure S3. Transient absorption spectra and kinetics for ReC0A in DMF outside cavity. (a) Transient absorption spectra at indicated delay times after 400 nm excitation. The solid lines in a) are fit to the TA spectra according to Equation (1) as described in SI 4.1. **(b)** Comparison of kinetic traces extracted from the measured and fitted spectra at 2050 and 2070 cm^{-1}. Shaded area of the kinetics from the fitted spectra represents error bars of one standard deviation of the residual.

SI 4.2. Extended Transient Transmission Spectra and Kinetics of On-Resonance Coupled ReC0A in the Cavity

Throughout this paper, normalized differential transmission is used to plot intracavity spectra, defined as $\Delta T = [I_{on} - I_{off}] / I_0$ where I_0 is the baseline of the incident IR light. To provide a more comprehensive view of the transient transmission dynamics, we present full

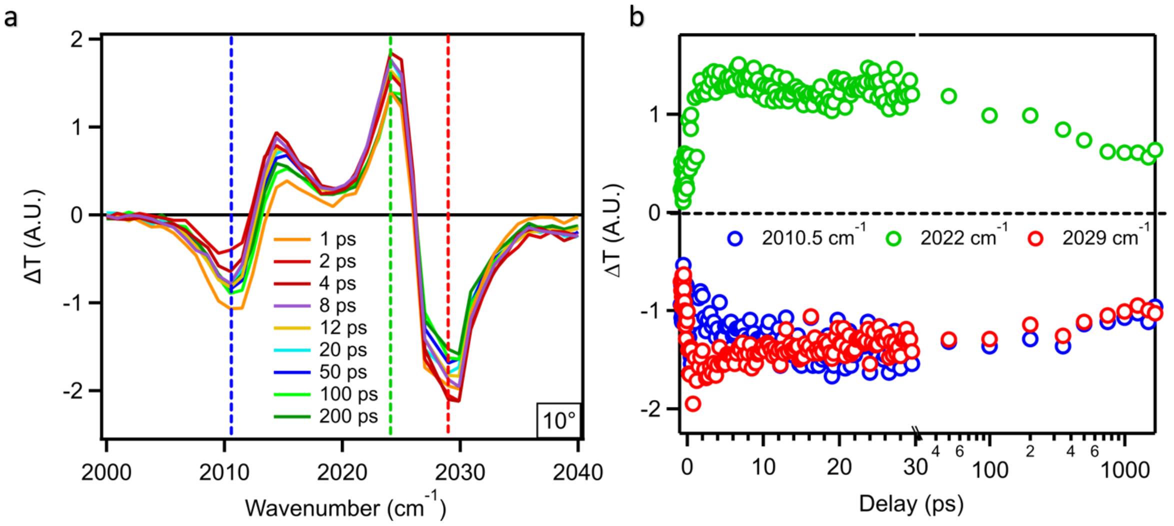

Figure S4. Extended on-resonance transient transmission of ReC0A in DMF inside a Fabry–Pérot microcavity following 400 nm excitation. (a) Full differential transmission spectra at selected pump–probe delays from 1 ps to 200 ps. Vertical dashed lines indicate frequencies at which kinetics are plotted. **(b)** Kinetic traces extracted at three probe frequencies (2010.5 cm^{-1} in blue circles; 2022 cm^{-1} in green circles; 2022 cm^{-1} in red circles).

time-resolved spectra of on-resonance coupled ReC0A inside the microcavity, spanning from 1 ps to 200 ps (Figure S4.a). Additionally, we include the kinetic traces of key spectral features up to 1.5 ns (Figure S4.b), extending beyond the 15 ps limit shown in the main text. The early-time behavior is discussed in the main text; the slower nanosecond-scale decay reflects bleach recovery back to the ground state.

SI.5 Computational Simulations

SI 5.1. Pump-probe Transmission

We modeled the pump-probe transmission spectrum of ReC0A in an IR microcavity using the Fabry-Perot transmission spectrum in the presence of an inhomogeneously broadened absorptive nonlinear dielectric medium. The pumped molecular ensemble is therefore characterized by its complex-valued index of refraction

$$n(\omega,\mathrm{t}) = n_R(\omega,\mathrm{t}) + i n_I(\omega,\mathrm{t}) \qquad (2)$$

where ω is the probed frequency and t is the pump-probe delay time. The real and imaginary parts of the refractive index of the system are obtained from the medium dielectric function $\epsilon(\omega,\mathrm{t}) = n^2(\omega,\mathrm{t})$, where $\epsilon(\omega,t) = \epsilon_R(\omega,t) + i\epsilon_I(\omega,t)$ and $\epsilon_R(\omega,t)$, $\epsilon_I(\omega,t)$ are the real and imaginary parts of $\epsilon(\omega,\mathrm{t})$ which we decompose into electronic ground and excited-state components

$$\epsilon(\omega,t) = \epsilon_G(\omega,t) + \epsilon_E(\omega,t) + \epsilon_b \qquad (3)$$

where ϵ_b is the background dielectric constant, and $\epsilon_G(\omega,t)$ and $\epsilon_E(\omega,t)$ are the contributions to the infrared susceptibility of the electronic ground state and excited molecules. The electrical susceptibilities of the molecules in the electronic ground and excited-state are parametrized by the time-dependent population fractions f_G, $f_E = 1 - f_G$, absorption coefficients A_G and A_E (which we assume to be equal to A for the sake of simplicity), homogeneous linewidths Γ_G, Γ_E, the vibrational resonance frequencies $\overline{\omega_G}$, $\overline{\omega_E(t)}$ and the inhomogeneous linewidths σ_G, $\sigma_E(t)$. The expressions for $\epsilon_G(\omega,t)$ and $\epsilon_E(\omega,t)$ are given

below.

$$\epsilon_G(\omega, t) = \int \frac{f_G A}{\omega^2 - \omega_G^2 - i\Gamma\omega_G} \phi(\omega_G - \overline{\omega_G}; \sigma_G) d\omega_G \qquad (4)$$

$$\epsilon_E(\omega, t) = \int \frac{f_E A}{\omega^2 - \omega_E^2 - i\Gamma\omega_E} \phi\big(\omega_E - \overline{\omega_E(t)}; \sigma_E(t)\big) d\omega_E \qquad (5)$$

where we introduced the Gaussian kernel $\phi(x; \sigma) = (2\pi\sigma^2)^{(-1/2)} e^{-x^2/2\sigma^2}$. Note the excited-state vibrational absorption frequency center and inhomogeneous linewidths are time-dependent quantities, whereas all other quantities including f_G and f_E are constant. We make the latter approximation because the electronic excited-state generation by the pump pulse occurs on a fs time scale that is much shorter than the pump-probe delay times investigated (1-20 ps). In addition, the molecular electronic excited-state decays on the nanosecond time scale, so the molecular ground and excited-state populations are essentially constant over the course of our experiment. As discussed in the main manuscript, our model assumes the molecular dynamics is essentially the same inside and outside the microcavity and this allows us to explain most of the experimental data. Therefore, we employ the following fits for the mean excited-state absorption frequency and Gaussian width obtained from measurements of the molecular system outside a microcavity

$$\overline{\omega_E(t)} = \omega_{E,0} + \big(\omega_{E,\infty} - \omega_{E,0}\big)\big[1 - e^{-t/\tau}\big] \qquad (6)$$

where $\omega_{E,0}$ and $\omega_{E,\infty}$ represent the excited state (Gaussian) band center frequency at $t = 0$ and at long times. A weighted smoothing spline was used to fit the experimental data of σ(t) (Gaussian width) versus delay time. A constant uncertainty of 0.75 was assigned to all data points, treating each point equally during the fitting process. The *UnivariateSpline* function from the *SciPy* library (Python) was employed for the fitting. The resulting spline fit had 8 degrees of freedom and was defined by 6 internal knots. The spline fit is described by a set of cubic polynomials between each pair of consecutive knot points, ensuring smoothness and continuity in both the first and second derivatives at the knots. The general functional form of the spline in each interval is given by:

$$S_i(t) = a_i + b_i(t - t_i) + c_i(t - t_i)^2 + d_i(t - t_i)^3 \text{ for } t_i \leq t \leq t_{i+1} \qquad (7)$$

where t_i represents the knot points, a_i, b_i, c_i, d_i are the coefficients for the cubic polynomial

in the ***i***-th interval and t is the delay time.

The real part of the molecular index of refraction $n_R(\omega, t)$ and the absorption coefficient $\alpha(\omega, t) = 4\pi\omega n_I(\omega, t)$ are employed in the Fabry-Perot transmission function to generate simulated time-dependent angle-resolved transmission spectra

$$T(\omega, \theta; t, L) = \frac{(1-R_M^2)e^{-\alpha(\omega,t)L(\theta)}}{1+R_M^2 e^{-2\alpha(\omega,t)L(\theta)} - R_M e^{-\alpha(\omega,t)L(\theta)+i\phi(\omega,\theta;\,t)}} \quad (8)$$

where L is the microcavity longitudinal length, R_M is the reflectivity of the mirrors (assumed to be equal), $L(\theta) = L/\cos(\theta)$, and $\phi(\omega, \theta; t) = 4\pi\, n_R(\omega, t)\, \omega\, L \cos(\theta)$.

The provided expressions allow us to obtain the equilibrium and nonequilibrium transmission spectrum of our molecular system inside a microcavity with a fixed length L. However, as mentioned in the main manuscript, microcavity length heterogeneity was a significant experimental factor, and we accounted for this in the simulations by performing an average of the reported transmission spectra (before and after pumping) over a normal distribution of lengths. This was obtained by a simple convolution of $T(\omega, \theta; t, L)$ with a Gaussian kernel $\phi(L - L_0;\ \sigma_L)$ where L_0 is the center length of the microcavity and σ_L corresponds to the root mean squared fluctuation of the microcavity length. Our final expression for the time-dependent transmission spectrum is given by

$$T(\omega, \theta; t) = \int T(\omega, \theta, t; L)\, \phi(\mathrm{L} - \mathrm{L}_0;\ \sigma_L)\, \mathrm{d}L \quad (9)$$

Experimental measured and simulated transmission spectra in transient and FTIR setup are shown in Figure S5. The measured upper and lower polariton peaks in the FTIR setup are at 2011 cm^{-1} and 2028 cm^{-1}, respectively, while in the transient setup, they are at 2012 cm^{-1} and 2026.5 cm^{-1}. The Rabi splitting shows a difference of 2.5 cm^{-1}. In addition, the linewidth measured in the FTIR setup is significantly broader than that in the transient setup. The broader linewidth in the FTIR setup arises because the larger probe beam size captures more cavity fluctuations. The simulation reproduces these results by assuming a 50 nm fluctuation in realized cavity lengths between the two setups, which aligns well with the fluctuation calculated from the free spectral ranges measured when light is focused onto different mirror positions.

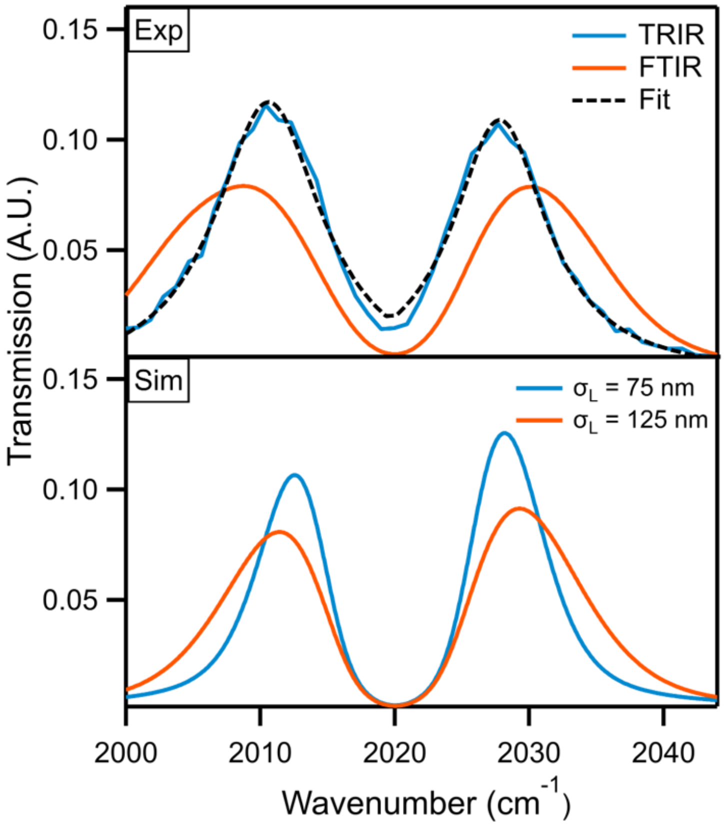

Figure S5. Rabi splitting difference induced by inhomogeneity. (Top) Comparison of the transmission spectra of the same ReC0A polariton sample measured by FTIR (blue trace) and transient IR setup (orange). The FTIR probe beam is significantly larger than the pulsed IR probe in the transient IR setup. **(Bottom)** Simulated linear transmission spectra of polariton modes with different cavity inhomogeneity as characterized by the root mean squared fluctuation of the microcavity length (σ_L). The simulated transmission spectra have been renormalized for better visualization.

A	ε_b	ω_{01}	Γ	σ_G	L_c	R_m	σ_0
350	2.04633	2020.6 cm^{-1}	6.9 cm^{-1}	4 cm^{-1}	40.396 μm	0.92	30 nm
f_e	$\omega_{E,0}$	$\omega_{E,\infty}$	τ				
0.01	2045 cm^{-1}	2063 cm^{-1}	3.4 ps				

Table S1. All parameters employed for our computations. A is the prefactor of the Lorentzian of the ground state, ε_b is the static permittivity of the solvent, ω_{01} is the 0-1 vibrational transition frequency, Γ and σ_G are the homogeneous and inhomogeneous broadening of the 0-1 transition, L_c is the simulated cavity length, R_m is the mirror reflectivity, σ_0 is the cavity length fluctuation (inhomogeneity), f_e is the percent of the molecules in the electronically excited state, $\omega_{E,0}$ and $\omega_{E,\infty}$ are the fitted center frequency of the excited state absorption at time zero and at long times relative to the fitted longitudinal relaxation time (solvation time) τ of the molecular dipole moment change induced by electronic excitation of the ReC0A molecule.

SI 5.2. Interpretation of transient transmission spectra inside microcavity

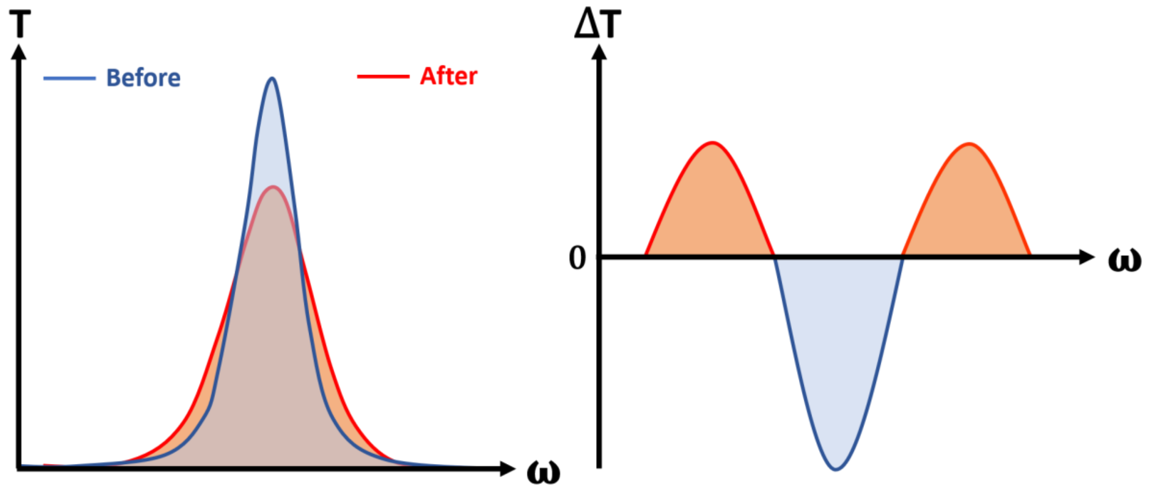

Figure S6. Simplified illustration of the absolute transmission (left) and transient transmission spectra (right). The change is exaggerated for clarity and the change is demonstrated to be symmetric for simplicity.

To understand the derivative spectra shape shown in Figure 4, we performed careful simulations with the methodology described in SI 5.1. The simulations reveal that the positive peak feature correlates strongly with the simulated microcavity mode linewidth. Within our tested range of incidence angles, an increase in the microcavity linewidth made the positive ΔT response more pronounced. After visible excitation, interactions between light, mirrors, molecules, and solvent molecules induce complex refractive index changes in both the sample and the mirrors. As a result, the absolute transmission of the targeted microcavity mode broadens. When comparing the transmission before and after excitation, a derivative-like feature emerges (Figure S6). The transmission amplitude change is subtle and unlikely to be perfectly symmetric, leading to skewed or twisted derivative shapes. Figure S6 provides an exaggerated illustration of how weak pump-induced broadening of a weakly coupled photon mode affects the corresponding microcavity transmission lineshape and its observed changes. It is important to note that the figure does not fully account for the time dependence of the excited state absorption band and the relaxation of the cavity mirrors following excitation, which could further slightly suppress or enhance ΔT.

SI 5.3. Perturbed free-induction decay

Our analysis of the perturbed free-induction decay (PFID) is simpler than our investigations of the positive time delay pump-probe transmission described above. Here, we considered only a single relevant photon mode corresponding to a fixed incidence angle in resonance with the targeted ReC0A vibrational mode following the standard approach of nonlinear spectroscopy formulated by Mukamel[1] (see also Hamm and Zanni[2] for further details). In a pump-probe measurement, the probe is typically weaker than the pump, allowing us to assume that the probe induces a linear response in the sample. Meanwhile, the pump field interacts twice with the sample, leading to a third-order response, which is the dominant nonlinear response given that the sample is assumed to be homogeneous and isotropic. Because the pulses are ultrafast, we can set $t_2 = 0$. In a heterodyne detection scheme, the primary focus is on the contribution to the detected intensity that arises from the interference between the probe's local oscillator field and the sample's response in the frequency domain:

$$S(w_3, t_1) - I_{LO} \propto 2\mathcal{R}e\left[\boldsymbol{E}_{LO}(w_3) \cdot \boldsymbol{E}_{sig}^{(3)}(w_3; t_1)\right] \tag{10}$$

There are three relevant pathways in the Liouville space The pathway that contains the contribution to the emitted signal from the pump-induced excited-state population can be summarized by the following scheme:

$$|g,0\rangle\langle g,0| \xrightarrow{probe} |g,1\rangle\langle g,0| \xrightarrow{\text{pump}(t_1)} \textstyle\sum_m |e,\widetilde{m}\rangle\langle g,0|$$
$$\xrightarrow{\text{pump}(t_2=0)} \textstyle\sum_{mn} |e,\widetilde{m}\rangle\langle e,\tilde{n}| \xrightarrow{\text{emission}(t_3)} \textstyle\sum_n |e,\tilde{n}\rangle\langle e,\tilde{n}| \tag{11}$$

Where the first index in the ket state refers to the electronic ground (g) or excited state (e). m and $\widetilde{m}$ represent mth vibrational state in ground and in the electronically excited state, respectively. When taking probe as the LO, the detected signal is given by:

$$\mathcal{R}e\left[\boldsymbol{E_{LO}}(w_3) \cdot \boldsymbol{E_{sig}^{(3)}}(w_3; t_1)\right]$$
$$\propto 2e^{-t_1/T_2}\mathcal{R}e\left\{\left[e^{-\mathrm{i}(\omega_{g0,g1}-\omega_3)t_1}\left[\frac{|\mu_{g0,g1}|^2 + \sum_m |\mu_{g0,e\widetilde{m}}|^2}{i(\omega_3-\omega_{g0,g1}) - \frac{1}{T_2}} - \sum_{mn} \frac{\mu_{g0,g1}\mu_{g0,e\widetilde{m}}\mu^*_{g0,e\tilde{n}}\mu_{e\tilde{n},e\widetilde{m}}}{i[\omega_3-(\omega_{e\widetilde{m}}-\omega_{e\tilde{n}})]-1/\tilde{T}_2}\right]\right]\right\} \tag{12}$$

The main difference relative to the analysis of PFID outside a microcavity is that the probe field at $\Delta t < 0$ generates LP and UP excitations originating from VSC, whose evolution is perturbed by the excitation of the system with the pump field. The probe-induced coherence consists of a superposition of LP and UP modes. The action of the pump can be characterized by employing the standard Feynman diagrams for LP and UP separately and the PFID signal written as a sum over all polaritonic contributions. In Liouville space, the pathway that has contribution to the emitted signal from the pump-induced excited-state population is represented by the following scheme:

$$|G,0,0_C\rangle\langle G,0,0_C| \xrightarrow{probe} |G,\mathrm{LP}\rangle\langle G,0,0_C| + |G,\mathrm{UP}\rangle\langle G,0,0_C|$$

$$\xrightarrow{\mathrm{pump}(t_1)} \sum_{i=1}^{N_M}\sum_{\tilde{m}_i} |e_i,\tilde{m}_i,0_C\rangle\langle G,0,0_C|$$

$$\xrightarrow{\mathrm{pump}(t_2=0)} \sum_{i=1}^{N_M}\sum_{\tilde{n}_i}\sum_{\tilde{m}_i} |e_i,\tilde{m}_i,0_C\rangle\langle e_i,\tilde{m}_i,0_C|$$

$$\xrightarrow{\mathrm{emission}(t_3)} |e_i,\tilde{m}_i,0_C\rangle\langle e_i,\tilde{m}_i,0_C| \qquad (13)$$

Here, in $|G,0,0_C\rangle$ represent the electronic state of all molecules with G meaning ground state, 0 meaning vibrational ground state and 0_C meaning no cavity photon presence. For the sake of simplicity, we assume polariton decay into reservoir modes (dark states) or via microcavity leakage is described by the decay rates γ_{LP} and γ_{UP}. A classical detector converts field intensity into currents and the detected signal originates from the real part of $\mathrm{E_{LO}}(\omega_3) \cdot E_{sig}{}^{(3)}(\omega_3;\mathrm{t}_1)$ is formulated as:

$$\mathcal{R}e[E_{LO}(w_3)\cdot E_{sig}^{(3)}(w_3;t_1)]$$

$$= \mathcal{R}e\left\{\left|V_{G_{00},G_{LP}}\right|^2 e^{-\mathrm{i}(\omega_{LP}-\omega_3)t_1} e^{-t_1/T_2^{LP}} \cdot \alpha(LP)\right\}$$

$$- \mathcal{R}e\left\{\left|V_{G_{00},G_{LP}}\right|^2 e^{-\mathrm{i}(\omega_{LP}-\omega_3)t_1} e^{-t_1/T_2^{LP}} \cdot \beta(LP)\right\}$$

$$+ LP \Leftrightarrow UP, \qquad (14)$$

where $\left|V_{G_{00},G_{LP}}\right|$ is the amplitude factor, α and β are simplified expression that represents the contribution of LP or UP to the oscillation, the last line means that the full expression

contains a similar term to the latter two where the LP is replaced by the UP. α and β is defined as:

$$\alpha \equiv \frac{\sum_{i=1}^{N_M}\sum_{m_i}\left|\mu_{G00,e_i\widetilde{m}_i0}\right|^2+\sum_{i=1}^{N_M}\sum_{m_i}\left|\mu_{G\mathrm{LP},e_i\widetilde{m}_i0}\right|^2}{i(\omega_3-\omega_{\mathrm{LP}})-\frac{1}{T_2^{\mathrm{LP}}}} \tag{15}$$

$$\beta \equiv \sum_{i=1}^{N_M}\sum_{mn}\frac{\mu_{G\mathrm{LP},e_i\widetilde{m}_i0}\cdot\mu_{G0,e_i\tilde{n}_i0}}{i[\omega_3-(\omega_{e_i\widetilde{m}_i}-\omega_{e_i\tilde{n}_i})]-1/\tilde{T}_2} \tag{16}$$

SI.6 Measured PFID outside and inside cavity

To confirm our understanding of PFID before investigating it inside the cavity, PFID outside the cavity (CaF_2 windows) was measured. Figure S7a shows the PFID signal measured outside the cavity for delays ranging from –4 ps up to just before time zero. At the longest negative delays (e.g. –4 ps, cyan trace) the oscillations are relatively rapid but of very small amplitude. As the pump–probe delay approaches zero, two clear trends emerge: first, the oscillation period lengthens (i.e. the beating slows down), and second, the overall oscillation amplitude grows markedly (see red trace at –1 ps). Both observations follow directly from the analytic form given in equation 12. There, the global prefactor e^{-t_1/T_2} dictates that the PFID amplitude should be maximal at $t_1 = 0$ and then decay exponentially at longer $|t|$, while the phase term $e^{-\mathrm{i}(\omega_{g0,g1}-\omega_3)t_1}$ produces a beating frequency equal to the detuning $\omega_{g0,g1} - \omega_3$, which remains effectively constant for large $|t|$ but, when combined with the sum over excited-state pathways in the second term, gives a slowly varying effective frequency near $t_1 = 0$. In other words, at early delays the full multi-level interference slows the apparent oscillation, and at later delays the single-mode detuning dominates, giving faster, low-amplitude beats—exactly as seen in Figure S7a. Figures S7b–d show single-frequency slices of the PFID kinetics at three representative probe wavenumbers (green circles), with the solid blue traces the result of a global fit to the full analytic form of equation 12. Panel (b) lies closest to the 2020 cm^{-1} CO stretch and consequently exhibits the slowest beat frequency and largest period, panel (c) at intermediate detuning shows a noticeably faster oscillation, and panel (d), furthest detuned from the 2020 cm^{-1} transition, displays the fastest beating. This clear trend—that increasing spectral separation from the molecular resonance leads to a higher oscillation frequency—is captured self-consistently by the detuning terms in equation 12, while the shared

decoherence time T_2 and resonance linewidth set the common decay envelope for all three kinetics.

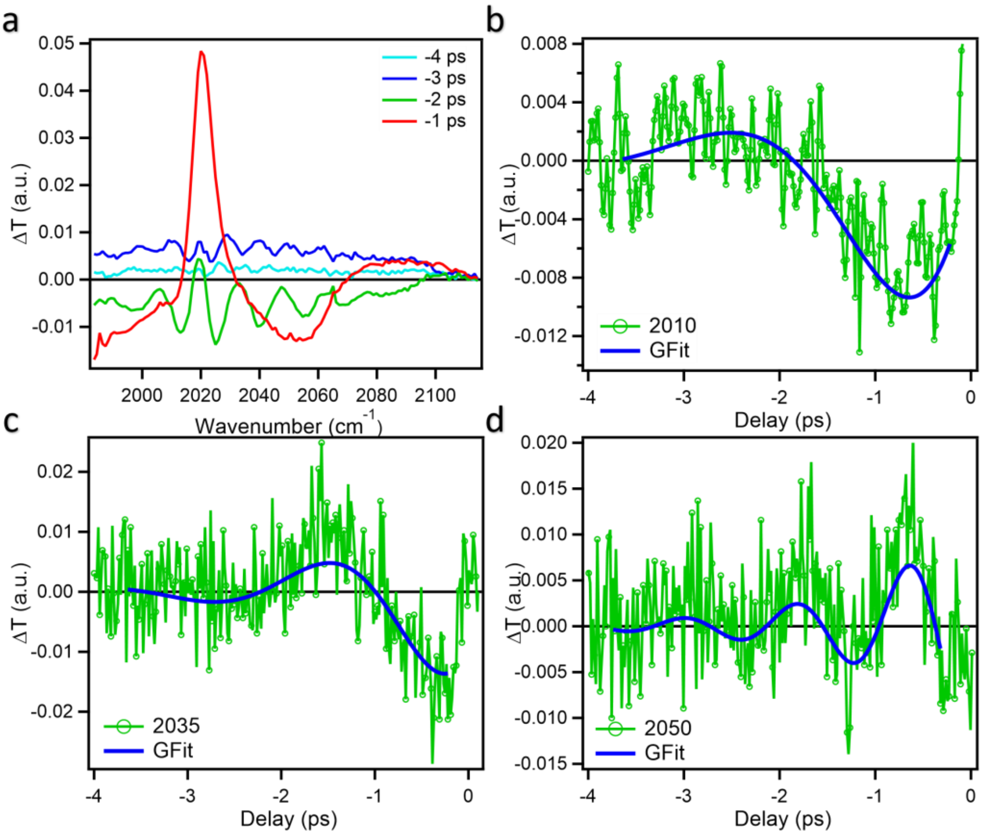

Figure S7. Transient free-induction decay (PFID) signals of ReC0A measured outside the cavity. Four different kinetics are globally fitted to equation 12. **(a)** PFID spectra recorded at four pump–probe delays (–4 ps: cyan; –3 ps: blue; –2 ps: green; –1 ps: red). **(b–d)** Selected PFID kinetics (green circles) at three probe wavenumbers: **(b)** 2020 cm^{-1} (on-resonance), **(c)** intermediate detuning, and **(d)** far-detuned. The further the probe is from the 2020 cm^{-1} CO stretch, the faster the observed beating. Solid blue lines are a simultaneous fit of all four kinetics to the full analytic form of equation 12, sharing a common coherence time T_2 and resonance parameters.

Figure S8 presents the transient measurements inside the cavity. The transient difference transmission spectra from -4 ps to -1 ps are shown in Figure S8a. The two probe frequencies selected for investigating the PFID inside the microcavity are 2010 cm^{-1} and 2029 cm^{-1}, targeting LP and UP kinetics, respectively. Notably, the observed PFID signals (represented by scattered dots) oscillate at 18.8 rad/ps (2998 GHz), while the model predicts a much slower oscillation essentially determined by the Rabi frequency at 3.7 rad/ps (600 GHz) shown in solid

lines. While the model correctly captures the signal outside the cavity, the dramatic difference in the order of magnitude inside the cavity suggests that the measured signal is not dominated by the molecular PFID feature. Further studies reveal that the observed feature is a convolution of three signals. The first contribution is the molecular PFID signal. Second, the dielectric mirror pairs can be excited by the visible and infrared pulses. The relaxation of the empty cavity takes about 1 ps, and the kinetics exhibit slow but pronounced oscillations (SI.7). Third, when DMF is present in the cavity without ReC0A (SI.7), the PFID signal shows very fast oscillations, similar to those observed with the sample in the cavity. DMF PFID oscillates at a frequency of (15.1 rad/ps, 2398 GHz). We conclude that in this system studied, the solute response is not the primary contributor to the observed signal at negative delay time.

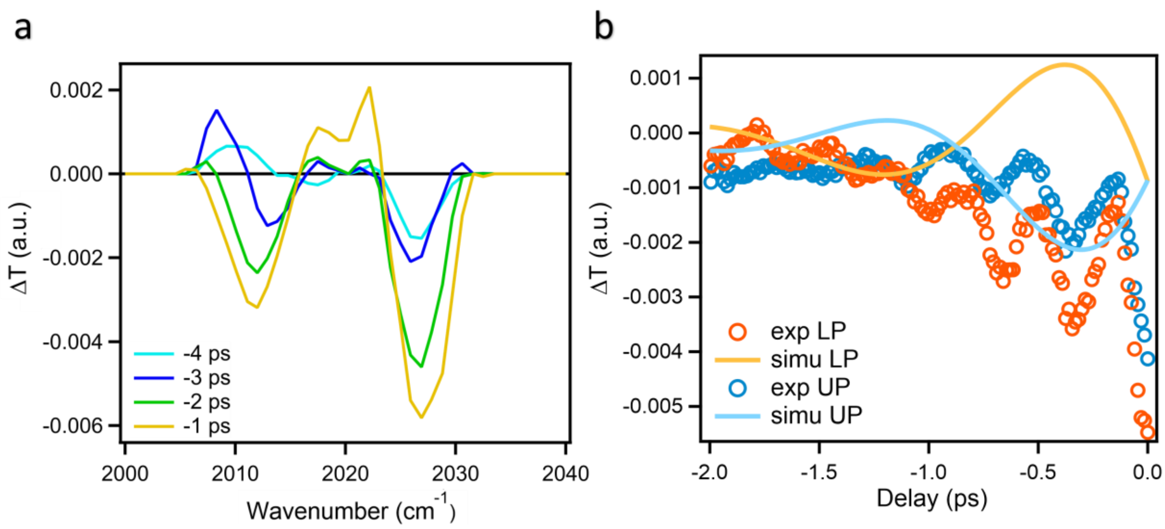

Figure S8. Transient transmission PFID of ReC0A spectra(a) and kinetics(b) inside the cavity. In panel b, experimental PFID kinetics inside the cavity at 2010 cm^{-1} (LP, orange circles) and 2029 cm^{-1} (UP, blue circles) are overlaid with the corresponding simulated traces (solid orange and blue lines). The measured oscillations occur significantly faster than the model predictions, indicating that the solute's response is not the dominant contributor to the cavity PFID signal.

SI.7 PFID with DMF and Acetonitrile (ACN) and Empty Cavity

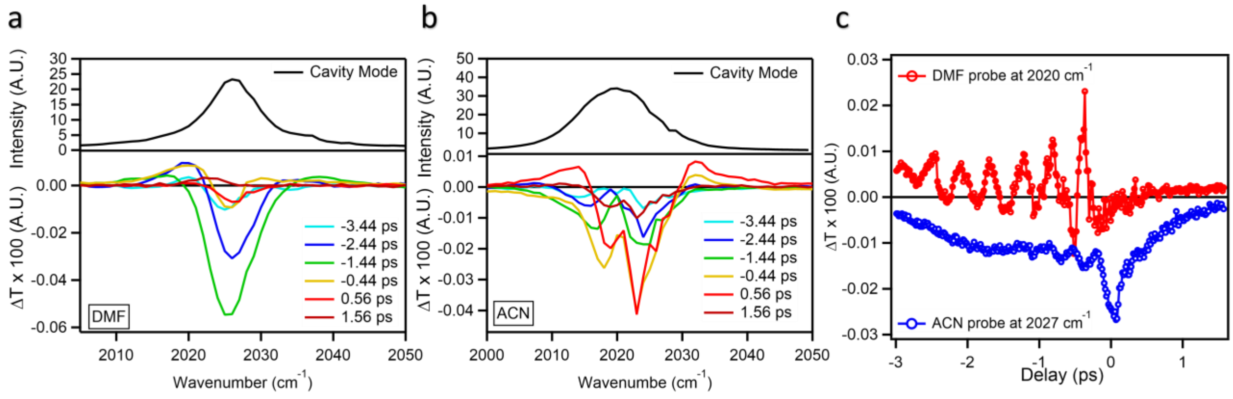

Figure S9. Transient transmission spectra and kinetics of Solvent PFID signal inside the cavity. Cavity modes are shown in black curves. **(a–b)** Upper panels: raw transient spectra of the cavity mode—angle-tuned to ~2020 cm⁻¹—for neat DMF (a) and ACN (b) at pump–probe delays from –3.44 ps (cyan) to +1.56 ps (brown). Lower panels: transient PFID spectra of these solvents (amplitude scaled ×100 to improve visibility). **(c)** Representative kinetic traces for DMF, probing at 2020 cm⁻¹ (red circles) and 2027 cm⁻¹ (blue circles). The oscillation frequencies do not match the predictions of our PFID model (Eq. 12).

We also investigated the perturbed free-induction decay (PFID) of DMF and ACN inside a Fabry–Pérot cavity. Although these solvents do not absorb in the visible range, we used the same 400 nm pump as in the ReC0A experiments. The transient spectra and kinetics are shown in Figure S9. In Figure S9a–b (upper panels), we plot the cavity modes—angle-tuned so that their resonance sits at ~2020 cm⁻¹—for neat DMF (a) and ACN (b) at pump–probe delays from –3.44 ps (cyan) to +1.56 ps (brown). Lower panels show the transient PFID spectra of the cavities with these solvents. Figure S9c then presents two representative kinetics for DMF (probe at 2020 cm⁻¹ in red circles and at 2027 cm⁻¹ in blue circles). While both DMF and ACN exhibit clear oscillatory signals, the corresponding frequencies could not be captured by our model (Equation 12); they do not match the simple difference between a solvent fundamental mode and the probe. Because the solvent PFID amplitudes are much weaker, the transmittance has been scaled by a factor of 100 to reveal the oscillations more clearly. Interestingly, the DMF oscillation in Figure S9c closely resembles that seen for ReC0A in DMF (Figure S8b), suggesting a common cavity-mediated origin. Moreover, there is a fast relaxation immediately after time zero that lasts up to ~1.5 ps, which we attribute to the cavity mirrors' response. Due to its small amplitude, we believe this will not affect our analysis in the main Stokes-shift

analysis.

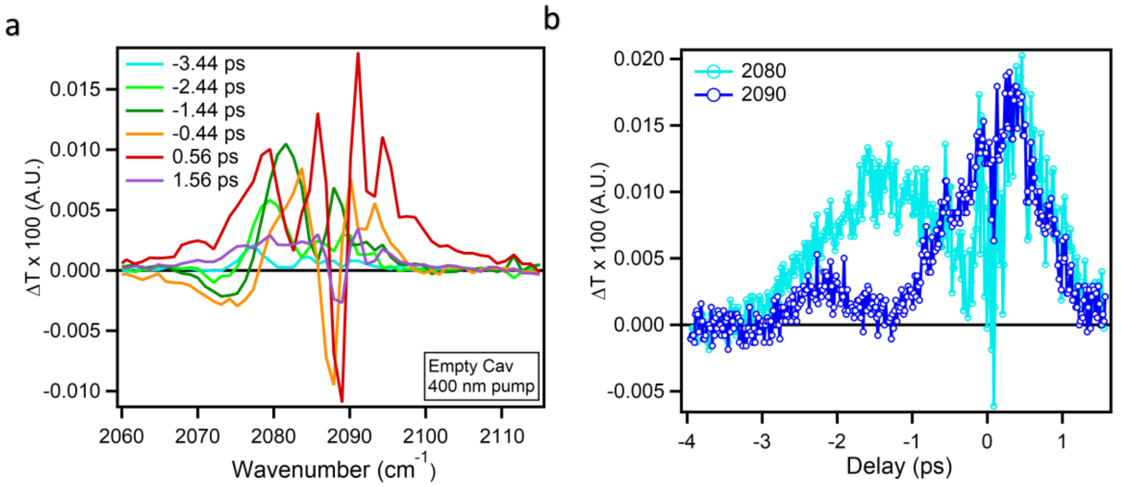

Figure S10. Transient transmission spectra of an empty cavity pumped by 400 nm pulse. (a) Raw pump–probe spectra of the bare cavity mode at selected delays from –3.44 ps (cyan) to +1.56 ps (magenta) **(b)** Kinetic traces of the transmission change at two probe frequencies (cyan and blue circles). Both traces exhibit an ultrafast modulation around time zero and a single exponential relaxation over ~1–2 ps. Transmittance are scaled by a factor of 100 for clear visibility.

Further studies, presented in Figure S10, revealed and justified the above statement that the dielectric mirrors forming the cavity are also being significantly affected by the pump, with modulation before time zero and a relaxation up till 1–2 ps. This suggests that the cavity not only filters the PFID signal but also that the dielectric mirrors and the cavity itself interact with the pump. The complete description of the PFID signal in the vibrational strong coupling regime is thus likely controlled by mirror nonlinearities[3], solvent response and contributions from reservoir modes (dark states), all of which play a significant role in shaping the observed signal. Moreover, as the delay approaches time zero, instrument response effects further complicate the analysis. Nevertheless, the amplitude of this mirror-mediated response remains small and does not interfere with or negate our main excited-Stokes-shift study and its conclusions.

References

1. Mukamel, S. (1995). *Principles of Nonlinear Optical Spectroscopy*.
2. Hamm, P., & Zanni, M. (2011). *Concepts and methods of 2D infrared spectroscopy*. Cambridge University Press.

3. Renken, S.; Pandya, R.; Georgiou, K.; Jayaprakash, R.; Gai, L.; Shen, Z.; Lidzey, D. G.; Rao, A.; Musser, A. J. Untargeted effects in organic exciton-polariton transient spectroscopy: A cautionary tale. *J Chem Phys* **2021**, *155* (15), 154701. DOI: 10.1063/5.0063173.
